# Transition metal-free oxidative and deoxygenative C–H/C–Li cross-couplings of 2*H*-imidazole 1-oxides with carboranyl lithium as an efficient synthetic approach to azaheterocyclic carboranes

**DOI:** 10.3762/bjoc.14.240

**Published:** 2018-10-12

**Authors:** Lidia A Smyshliaeva, Mikhail V Varaksin, Pavel A Slepukhin, Oleg N Chupakhin, Valery N Charushin

**Affiliations:** 1 Ural Federal University, 19 Mira Str., 620002 Ekaterinburg, Russia; 2 Institute of Organic Synthesis, Ural Branch of the Russian Academy of Sciences, 22 S. Kovalevskaya Str., 620041 Ekaterinburg, Russia

**Keywords:** carboranes, C–H functionalization, C–H/C–Li cross-coupling, 2*H*-imidazole 1-oxide, nucleophilic substitution of hydrogen (S_N_^H^)

## Abstract

The direct C–H functionalization methodology has first been applied to perform transition metal-free C–H/C–Li cross-couplings of 2*H*-imidazole 1-oxides with carboranyllithium. This atom- and step-economical approach, based on one-pot reactions of nucleophilic substitution of hydrogen (S_N_^H^) in non-aromatic azaheterocycles, affords novel imidazolyl-modified carboranes of two types (*N*-oxides and their deoxygenative analogues), which are particularly of interest in the design of advanced materials.

## Introduction

Dicarbadodecaboranes (carboranes) are known to be 3D polyhedral clusters with a special type of structural organization, relative thermal and chemical stabilities, as well as unique physicochemical properties [1–3]. The functional derivatives of carboranes, in particular heterocyclic ones, are undeniably of increased interest in the chemistry of organoboron compounds due to wide opportunities to use these boron-enriched substances as diagnostic tools for tumor radio imaging [4–7], promising agents for boron neutron capture therapy (BNCT) of cancer [8–12], as well as agonists and antagonists of biological receptors [13–17], etc. In addition, azaheterocyclic carboranes are actively used as ligands in the synthesis of metal complexes of various architectures, possessing catalytic activity [18–21], as well as unique photophysical properties [22–26]. Thus, the development of effective approaches to azaheterocyclic carboranes is currently considered to be one of the most important synthetic challenges.

At present, there are three main synthetic strategies in the design of promising heterocyclic carboranes: (i) condensation of decaborane (B_10_H_14_) with substituted acetylenes [27–30], (ii) carboryne-based cycloaddition reactions [31–33], and (iii) C–X/C–M cross coupling of halogenated azaheterocycles (X = Br, Cl, F) with carborane organometallic derivatives (M = Li, Cu) [11,22,34–35]. Despite the fact that the latter synthetic strategy is in common use, it has significant limitations. In particular, the preliminary functionalization of heterocyclic substrates is required to apply this cross-coupling methodology.

Thus, the development of pot-, atom- and stage-economical (PASE) [36–38] methods leading to novel C-modified *ortho*-carboranes, as well as elucidation of scope and mechanistic features of these transformations, is one of the crucial steps to obtain promising boron-enriched heterocyclic ensembles and functional materials based on them. An alternative approach to exploit the C–X/C–M cross-coupling reactions, leading to heterocyclic boron clusters, is based on the C–H/C–M coupling strategy. One of the ways to realize these cross couplings is the transition metal-free methodology for direct C–H functionalization of azaheterocyclic substrates [39–42], which can be carried out by using S_N_^H^ reactions (nucleophilic substitution of hydrogen) [43–48]. The S_N_^H^ methodology corresponds to the basic principles of green chemistry [49–53], and it is now considered to be one of the most valuable approaches to functionally substituted azaheterocycles, since these transformations can be performed without any catalysis by transition metals, neither they need preliminary introduction of good leaving groups into heterocyclic substrates.

It is known that S_N_^H^ reactions have successfully been applied in the chemistry of π-deficient (hetero)aromatic systems as an effective and useful synthetic approach [43–48]. These transformations have been used for the synthesis of various mono-, di-, and triazinyl-modified carboranes [54–56]. At the same time, a limited number of protocols describe application of S_N_^H^ reactions to functionalize non-aromatic heterocycles. In particular, only few examples of S_N_^H^ reactions are available in the chemistry of 2*H*-imidazole 1-oxides [57–60] that are of interest as promising pharmacoactive azaheterocyclic compounds [61–65].

In this paper, we wish to report the first examples of the S_N_^H^ methodology for the synthesis of new heterocyclic carboranes by means of direct C–H/C–Li coupling of non-aromatic 2*H*-imidazole 1-oxides with carboranyllithium. A growing interest in bifunctional compounds, bearing both the pharmacoactive imidazole motif and a polyhedral boron-enriched scaffold, is likely to be due to unique properties of these organoboron compounds and materials based on them.

## Results and Discussion

2*H*-Imidazole 1-oxides are known to be non-aromatic heterocyclic compounds, bearing the electrophilic center C(5)–H, which is active for interaction with nucleophilic reagents. This feature enables one to carry out the direct nucleophilic functionalization of the C(sp^2^)–H bond, thus leading to novel C(5)-modified 2*H*-imidazoles. We have established that carboranyllithium, generated in situ from 1,2-dicarba-*closo*-dodecaborane [66], can be involved in the above mentioned C–H/C–Li coupling reactions successfully as a nucleophilic partner.

The direct transition metal-free C–H/C–Li cross-coupling reactions of 2*H*-imidazole 1-oxides **1a**–**d** with **2** have been found to result in the novel carboranes **4a**–**d** and **5a**–**d** of various architectures. These transformations are able to be considered as nucleophilic substitution of hydrogen (S_N_^H^) in non-aromatic *N*-oxides of 2*H*-imidazoles **1a**–**d**. It has also been shown that the S_N_^H^ reactions can be realized via either an "addition–elimination" S_N_^H^(AE) mechanism, or through an "addition–oxidation" S_N_^H^(AO) scheme to form imidazolyl carbonanes **4a**–**d** and **5a**–**d**, respectively.

In accordance with the current S_N_^H^ concept, the first step of both protocols, S_N_^H^(AE) and S_N_^H^(AO), involves a reversible formation of unstable anionic σ^H^-adducts **3(a**–**d)-OLi** as a result of nucleophilic attack of carboranyllithium **2** at the CH=N^+^–O^−^ bond of 2*H*-imidazole 1-oxides **1a**–**d**. The second step can be carried out in both eliminative and oxidative versions, the structure of the final carboranylated 2*H*-imidazoles **4a–d** and **5a**–**d** being controlled by the reaction conditions used.

**Scheme 1:**
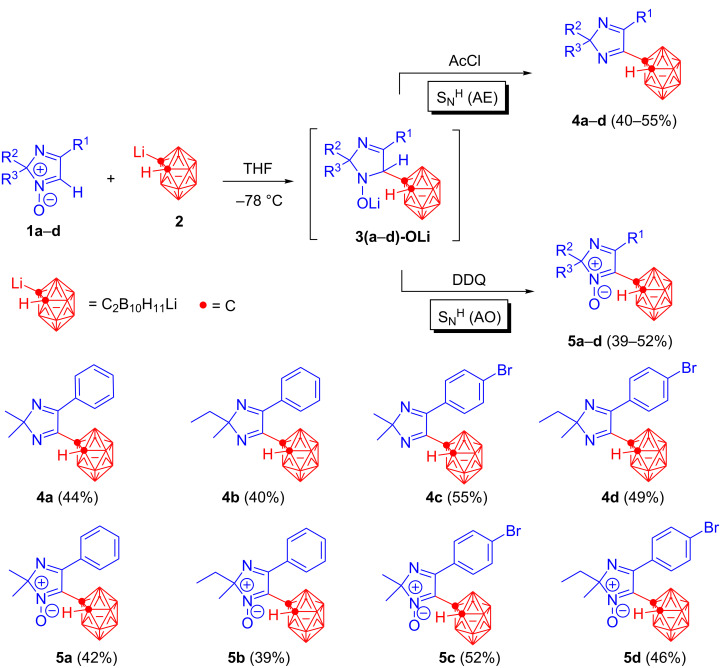
C–H/C–Li cross-coupling reactions of 2*H*-imidazole 1-oxides **1a**–**d** and carboranyl lithium **2**. The reactions were carried out in accordance with the optimized coupling conditions according to the "addition–elimination" S_N_^H^(AE) or "addition–oxidation" S_N_^H^(AO) pathways (Table 1 and Table 2).

In order to accomplish the nucleophilic substitution of hydrogen according to the "addition–elimination" scheme S_N_^H^(AE), the key factor has been shown to be the presence of a deoxygenating reagent in the reaction mixtures, containing the σ^H^-adducts **3(a**–**d)-OLi**. Indeed, it has been found that addition of acylating agents to intermediates **3(a**–**d)-OLi** facilitates elimination of hydrogen from the σ^H^-adducts along with the oxygen-containing fragment from the N^+^–O^−^ moiety to form imidazolyl-substituted carboranes **4a**–**d** with the loss of the *N*-oxide function (Scheme 1).

In order to find out the optimal reaction conditions for the S_N_^H^(AE) pathway, affording the maximum yields of the target products **4**, the effects of various factors (type of acylating agents, exposure time for which the reaction mass is kept after mixing 2*H*-imidazole *N*-oxide **1** with carboranyllithium **2** and quenching with an acylating agent, as well as the temperature regime, at which the deoxygenative agent is added) have been studied. The cross-coupling reaction of 2,2-dimethyl-4-phenyl-2*H*-imidazole 1-oxide (**1a**) with carboranyllithium **2** was chosen as a model reaction. It has finally been found that the best yield of **4a** is achieved using AcCl as a deoxygenative agent at room temperature with stirring of the resulted reaction mixture for 15 min (Table 1, entry 11). Thus, a number of novel heterocyclic carboranes **4a**–**d** have been synthesized in 40–55% yields following the optimized reaction conditions (Scheme 1).

**Table 1:** Optimization of the reaction conditions for the C–H/C–Li cross-coupling of 2*H*-imidazole 1-oxides **1a**–**d** with carboranyllithium **2** according to the "addition–elimination" protocol^a^.

entry	temperature, °C^b^	exposure time (min)^c^	acylating agent	yield of **4a** (%)

1	−78	15	Ac_2_O	9
2	−78	30	Ac_2_O	9
3	0	15	Ac_2_O	17
4	0	30	Ac_2_O	17
5	rt	15	Ac_2_O	28
6	rt	30	Ac_2_O	27
7	−78	15	AcCl	12
8	−78	30	AcCl	12
9	0	15	AcCl	23
10	0	30	AcCl	23
**11**	**rt**	**15**	**AcCl**	**43**
12	rt	30	AcCl	40
13	−78	15	TFAA	7
14	−78	30	TFAA	7
15	0	15	TFAA	14
16	0	30	TFAA	14
17	rt	15	TFAA	18
18	rt	30	TFAA	18

^a^The reaction was carried out in dry THF using 2*H*-imidazole 1-oxide **1a** (1.1 equiv), carboranyllithium **2** prepared from *o*-carborane (1.0 equiv) and *n*-BuLi (1.1 equiv) at −78 °C. ^b^The temperature at which the acylating agent was added. ^c^Between addition of 2*H*-imidazole 1-oxide **1a** to carboranyllithium **2** and quenching with the acylating agent.

In case of the "addition–oxidation" protocol realization for the S_N_^H^ (AO) reactions, an oxidative agent to convert the σ^H^-adducts **3(a**–**d)-OLi** into the corresponding imidazolyl carboranes **5a**–**d** with retention of the *N*-oxide function in the imidazole moiety, has been found to play a key role. It should be noted that optimization of the reaction conditions for oxidative C–C couplings has been carried out by using the model reaction of 2,2-dimethyl-4-phenyl-2*H*-imidazole 1-oxide (**1a**) with carboranyllithium **2**. The experiments performed have shown effects of the used oxidants, temperature regime, and exposure time after addition of an oxidant into the reaction mixture. As a result, the optimal conditions have been found to involve the use of DDQ as oxidant and refluxing of the reaction mixture in argon atmosphere for 1 h (Table 2, entry 12). It has also been observed that further increase in the exposure time does not improve yields (39–52%) of the target carboranyl-substituted imidazole 1-oxides **5a**–**d** (Scheme 1). Besides compounds **5a**–**d**, the formation of their deoxygenated analogues **4a**–**d** has been shown to take place in trace amounts under the optimized reaction conditions. The latter are supposed to be derived from elimination of hydrogen and the oxygen-containing moiety from the corresponding σ^H^-adducts.

**Table 2:** Optimization of the reaction conditions for the C–H/C–Li cross-coupling of 2*H*-imidazole 1-oxides **1a**–**d** with carboranyllithium **2** according to the "addition–oxidation" protocol^a^.

entry	oxidant	temperature, °C^b^	exposure time (min)^c^	yield of **5a** (%)

1	–	Rt	30	trace
2	–	Rt	60	trace
3	–	40	30	5
4	–	40	60	7
5	–	reflux	30	9
6	–	reflux	60	12
7	DDQ	Rt	30	7
8	DDQ	Rt	60	7
9	DDQ	40	30	9
10	DDQ	40	60	11
11	DDQ	reflux	30	35^d^
**12**	**DDQ**	**reflux**	**60**	**42**^d^
13	DDQ	reflux	120	42^d^
14	*p*-chloranyl	rt	60	5
15	*p*-chloranyl	40	60	7
16	*p*-chloranyl	reflux	30	17
17	*p*-chloranyl	reflux	60	31
18	*p*-chloranyl	reflux	120	30

^a^The reaction was carried out in dry THF using 2*H*-imidazole 1-oxide **1a** (1.1 equiv), carboranyllithium **2** prepared from *o*-carborane (1.0 equiv) and *n*-BuLi (1.1 equiv) at −78 °C. ^b^The temperature at which the reaction mixture was stirred after addition of oxidant. ^c^Exposure time after addition of oxidant to the reaction mixture. ^d^Imidazolyl carborane **4a** was isolated in trace amounts.

It is worth mentioning that the C–H/C–Li coupling reactions of 2*H*-imidazole 1-oxides **1c**,**d** with carboranyllithium **2** have been found to result in the formation of *p*-bromophenyl derivatives **4c**,**d** and **5c**,**d**, which are of particular interest as valuable synthons for further modifications, for instance by means of transition metal-catalyzed C–Br/C–M (M = Li, Mg, Zn, etc.) and C–Br/C–H cross-couplings reactions leading to more complex organic compounds.

**Figure 1:**
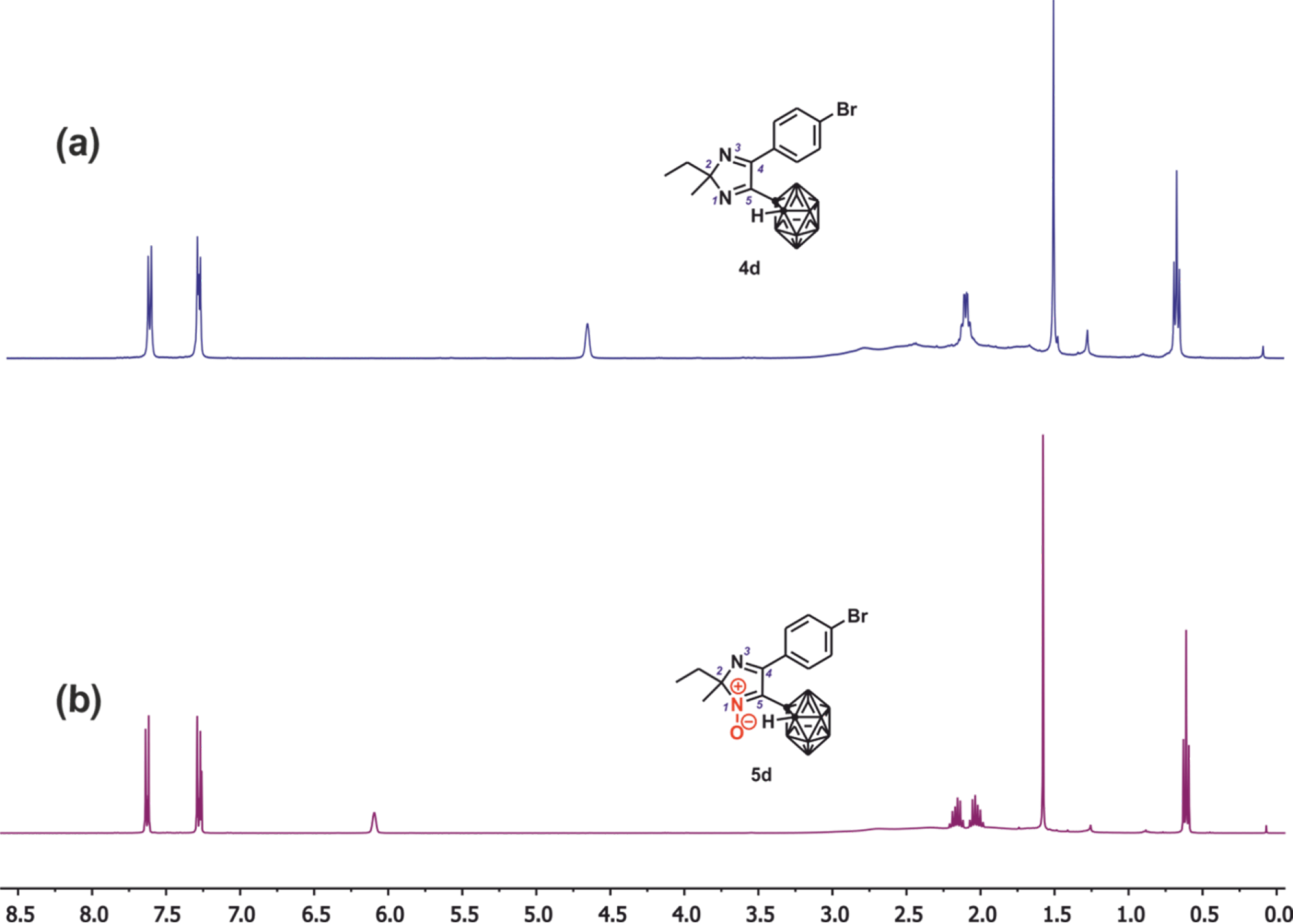
The ^1^H NMR spectra of 1-(5-(4-bromophenyl)-2-ethyl-2-methyl-2*H*-imidazol-4-yl)-1,2-dicarba-*closo*-dodecaborane (**4d**) and 1-(4-(4-bromophenyl)-2-ethyl-2-methyl-1-oxido-2*H*-imidazol-5-yl)-1,2-dicarba-*closo*-dodecaborane (**5d**) in CDCl_3_ at 295 K.

Mono-substituted imidazolyl carboranes **4a**–**d** and their *N*-oxide analogues **5a**–**d** were characterized by the data of elemental analysis, IR, NMR spectroscopy (^1^H, ^13^C{^1^H} (APT), ^11^B, ^11^B{^1^H} experiments), and mass spectrometry. In the IR spectra of **4a**–**d** and **5a**–**d**, there are absorption bands corresponding to the stretching vibrations of carborane B–B atoms (ν 713–721 cm^−1^), B–H (ν 2561–2605 cm^−1^) and C–H (ν 3026–3075 cm^−1^). In the ^1^H NMR spectra of **4a**–**d** and **5a**–**d** the resonance signals of both carborane and imidazole fragments were observed. Typical ^1^H NMR spectra of carboranyl-substituted imidazoles and their *N*-oxides (**4d** versus **5d**) are shown in Figure 1. In these spectra, the characteristic signals of C-substituted carborane are registered as two sets, corresponding to C_Carb_–H and B–H protons. The chemical shift of unsubstituted carborane C_carb_–H proton resonance has been shown to depend on the presence of the N^+^–O^–^ moiety in the imidazole ring. In particular, a broadened singlet of the C_carb_–H proton is exhibited at δ 4.63–4.58 ppm in the ^1^H NMR spectra of **4a**–**d**, while in the spectra of their *N*-oxide analogues **5a**–**d** the corresponding singlet is observed at δ 6.09–6.03 ppm. Such difference in chemical shifts is likely to be due to the effect of the imidazole N^+^–O^–^ group oxygen. Carborane B–H protons are observed as 10 H broadened multiplets at δ 3.08–1.61 ppm. The proton resonances of C(2)-alkyl and C(4)-aryl substituents are observed in the appropriate fields: at δ 2.17–0.61 ppm and δ 7.67–7.22 ppm, respectively.

In the ^13^C NMR spectra, one can also see the difference in chemical shifts for carbon-13 resonance signals of imidazoles **4a**–**d** and their *N*-oxide analogues **5a**–**d**. Indeed, in the ^13^C NMR spectra of **4a**–**d** the carbon-13 resonances of organoboronic C_carb_–H fragments are observed at δ 61.75–61.47 ppm, while in the spectra of their *N*-oxides **5a**–**d** the corresponding signals are exhibited at δ 57.64–57.63 ppm. The carbon resonances associated with the imidazole ring have been recorded at δ 69.36–69.12 ppm and at δ 65.66–65.41 ppm in the ^13^C NMR spectra of compounds **4a**–**d** and **5a**–**d**, respectively. In the mass spectra of carboranylimidazoles the corresponding molecular ion [M]^+^ peaks have also been registered. It should be mentioned that [M]^+^ values, observed for the *N*-oxide series of compounds **5a**–**d**, proved to differ from those registered for the deoxygenated analogues **4a**–**d** by atomic oxygen mass (16 amu).

**Figure 2:**
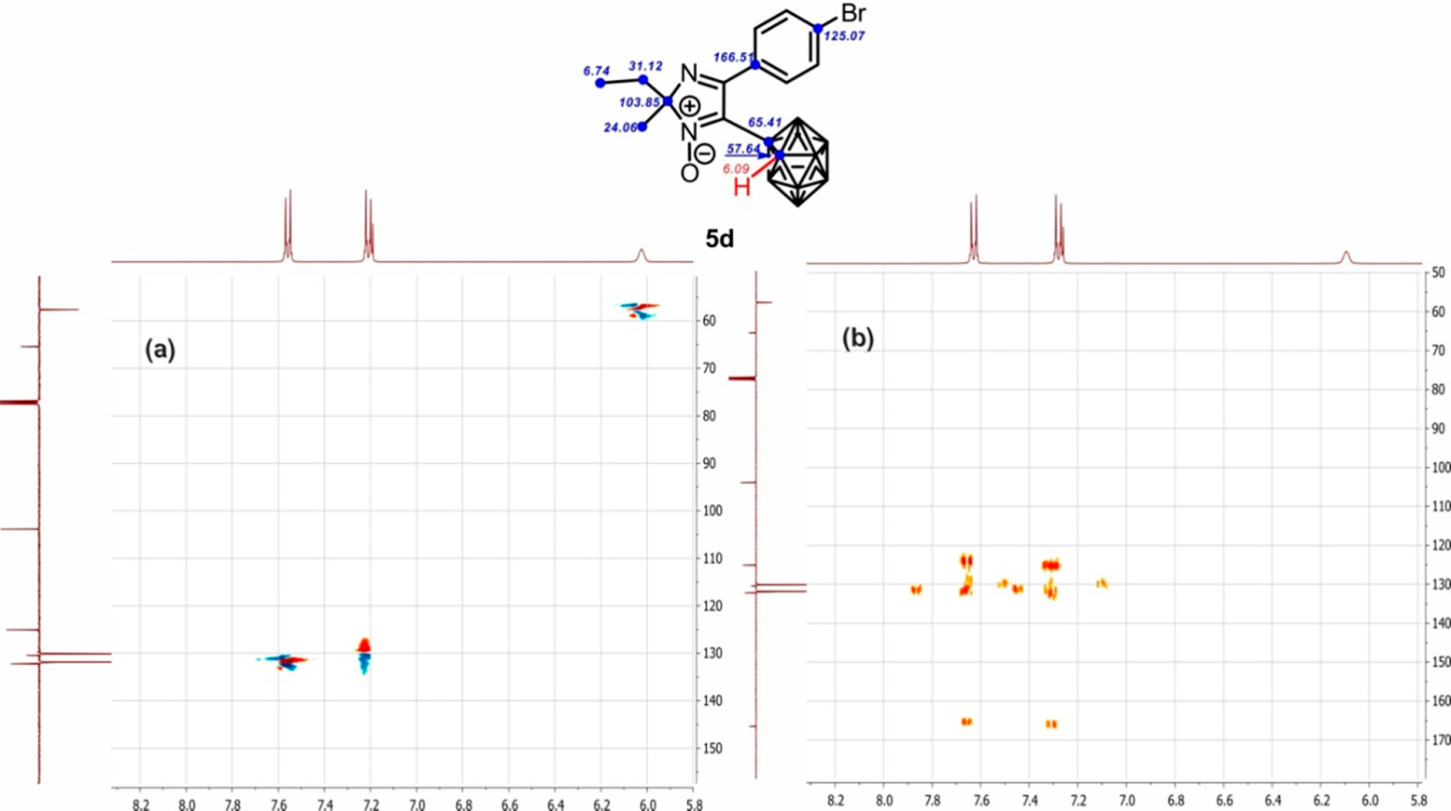
Fragment of the 2D ^1^H–^13^C{^1^H} HSQC (a) and HMBC (b) spectra of imidazolyl carborane **5d** in CDCl_3_ at 295 K (the whole spectra are shown in Supporting Information File 1).

The novel imidazolyl carboranes **4a**–**d** and **5a**–**d** obtained are considered to be complex *closo*-cluster structures formed by polyboron and heterocyclic scaffolds linked to each other through the C–C bond. In order to correlate the resonance signals with the assumed structure unambiguously, two-dimensional NMR correlation ^1^H–^13^C spectra, showing direct (HSQC) and distant (HMBC) spin–spin constants, have been recorded for compound **5d** (Figure 2). The presence of the cross-peak {6.09, 57.64} in the HSQC spectrum of **5d** and absence of any signals in these regions in the HMBC spectrum enables one to state that these signals belong to proton (δ 6.09 ppm) and carbon (δ 57.64 ppm) resonances of the C_Carb_–H fragment. Also, the 2D spectra allow one to distinguish the carbon resonances of 2*H*-imidazole methyl (δ 24.06 ppm) and ethyl (δ 6.74 and 31.12 ppm) groups. In addition, the interaction observed in the HMBC spectra of quaternary carbons with protons of methyl and ethyl groups through the long-range constants allows the signal at δ 103.85 ppm in the ^13^C NMR spectra of **5d** to be attributed to C-2 of the imidazole ring.

**Figure 3:**
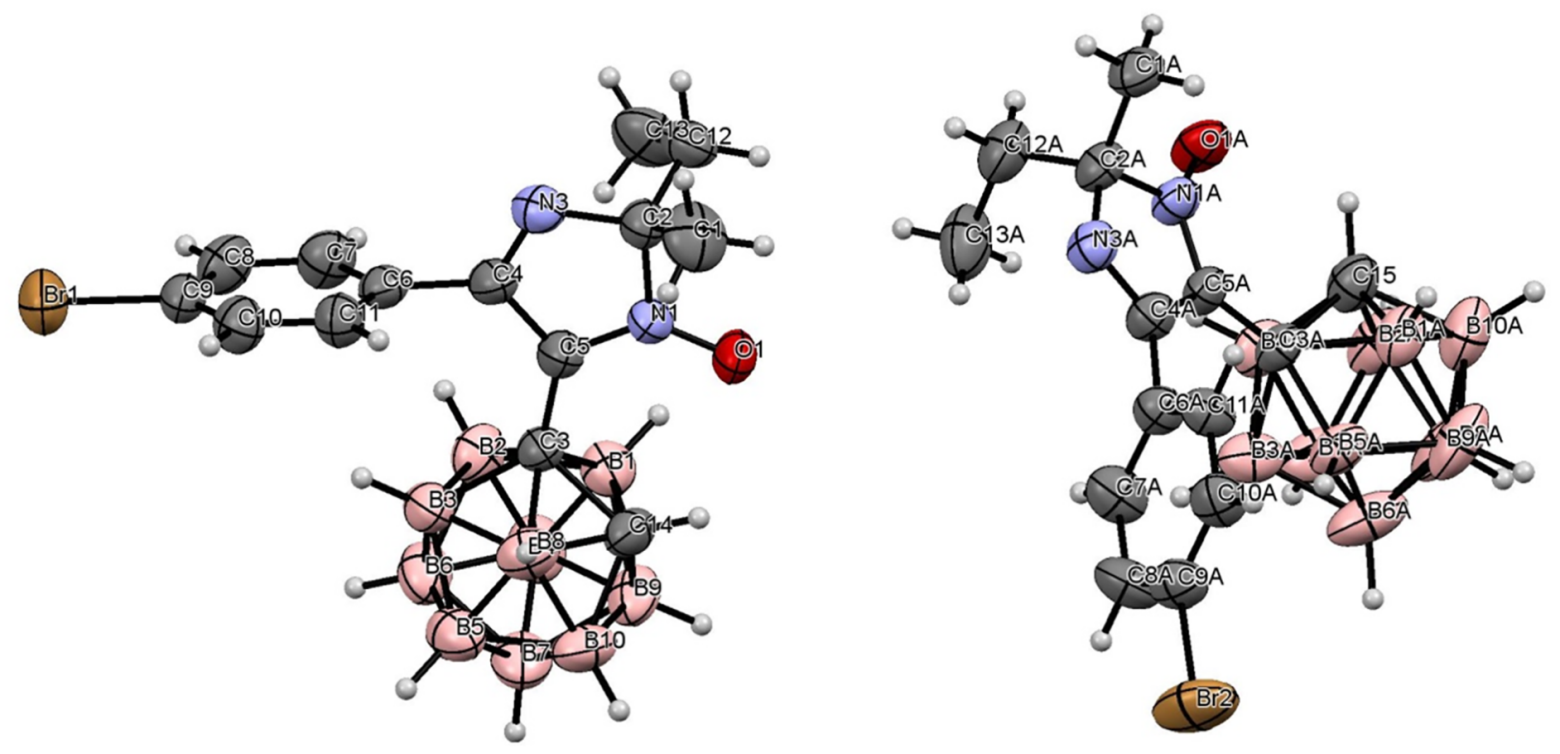
Molecular structure of **5d**. Selected bond distances (Å) and angles (deg) for molecule 1: C(3)–C(14), 1.665; C(3)–C(5), 1.491; C(5)–N(1), 1.338; C(5)–C(4), 1.472; Br(1)–C(9), 1.910; O(1)–N(1), 1.281; N(3)–C(4), 1.30; N(3)–C(2), 1.45; C(2)–C(1), 1.54; C(2)–C(12), 1.53; C(6)–C(4), 1.499; C(4)–N(3)–C(2), 108.2; O(1)–N(1)–C(2), 120.3; O(1)–N(1)–C(5), 130.0; C(5)–C(3)–C(14), 116.6; C(12)–C(2)–N(1), 108.4; C(1)–C(2)–N(3), 111.4; C(1)–C(2)–C(12), 113.8; for molecule 2 “A”: C(3A)–C(15), 1.65; C(3A)–C(5A), 1.500; C(5A)–N(1A), 1.326; C(5A)–C(4A), 1.468; Br(2)–C(9A), 1.904; O(1A)–N(1A), 1.267; N(3A)–C(4A), 1.300; N(3A)–C(2A), 1.441; C(2A)–C(1A), 1.523; C(2A)–C(12A), 1.532; C(6A)–C(4A), 1.491; C(4A)–N(3A)–C(2A), 108.6; O(1A)–N(1A)–C(2A), 120.4; O(1A)–N(1A)–C(5A), 130.4; C(5A)–C(3A)–C(15), 116.5; C(12A)–C(2A)–N(1A), 107.1; C(1A)–C(2A)–N(3A), 113.0; C(1A)–C(2A)–C(12A), 112.0.

The structure of imidazolyl carborane **5d** has also been proved by the X-ray analysis (Figure 3). The single crystals were obtained by slow evaporation of imidazolyl carborane **5d** from a mixture of CH_2_Cl_2_/heptane, 8:2. According to the XRD data, two independent molecules have been found to be crystallized in the *P*12_1_1 chiral space group of the monoclinic system. General views for molecules 1 and 2 are shown in Figure 3, atoms of the molecule 2 being labeled with the additional index “A”. Both independent molecules proved to be characterized by the (*R*)-configuration of the C(2)-chiral center and the appropriate bond and angle distances. Both heterocyclic rings have been found to be planar with 4-BrC_6_H_4_ substituent being turned towards the heterocyclic fragments, thus forming the angles of 74° and 80°, respectively. In the crystals of molecule 1, the 2*H*-imidazole 1-oxide fragment is disordered into two positions with occupancy coefficient of 0.8/0.2. The minor disordered moiety has been confirmed to be in the (*S*)-configuration. Also it is worth mentioning that no shortened contacts have been observed in the crystals.

## Conclusion

The direct C(sp^2^)–H functionalization methodology has first been applied to design the synthesis of polyhedral boron *closo-*clusters. Novel heterocyclic carboranes have been obtained through a one pot-, atom- and stage-economical approach, based on nucleophilic substitution of hydrogen (S_N_^H^) in 2*H*-imidazole 1-oxides by action of carboranyllithium, generated in situ from commercially available *o*-carborane. It has also been found that the S_N_^H^(AE, «addition–elimination») scheme leads to carboranyl-substituted 2*H*-imidazoles, while alternative S_N_^H^(AO, «addition–oxidation») results in the corresponding *N*-oxide analogues. In summary, novel organoelement bifunctional ensembles, bearing heterocyclic and carborane moieties, which are of particular interest in the design of advanced materials, have been obtained in good yields.

## Supporting Information

File 1 Experimental procedures, characterization data, copies of the ^1^H, ^13^C, ^11^B NMR spectra and X-ray diffraction studies.
