# Supplementary material for: Transition metal-free oxidative and deoxygenative C–H/C–Li cross-couplings of 2H-imidazole 1-oxides with carboranyl lithium as an efficient synthetic approach to azaheterocyclic carboranes
Source: Beilstein J Org Chem. 2018 Oct 12;14:2618–26. doi: 10.3762/bjoc.14.240 (PMC6204773; doi:10.3762/bjoc.14.240)
Supplement: File 1 — Experimental procedures, characterization data, copies of the 1H, 13C, 11B NMR spectra and X-ray diffraction studies. [file Beilstein_J_Org_Chem-14-2618-s001.pdf]

Supporting Information

for

Transition metal-free oxidative and deoxygenative C–H/C–Li cross-couplings of 2*H*-imidazole 1-oxides with carboranyl lithium as an efficient synthetic approach to azaheterocyclic carboranes

Lidia A. Smyshliaeva[1], Mikhail V. Varaksin[1,2], Pavel A. Slepukhin[1,2], Oleg N. Chupakhin[*,1,2] and Valery N. Charushin[1,2]

Address: [1]Ural Federal University, 19 Mira Str., 620002 Ekaterinburg, Russia and [2]Institute of Organic Synthesis, Ural Branch of the Russian Academy of Sciences, 22 S. Kovalevskaya Str., 620041 Ekaterinburg, Russia

E-mail: Oleg N. Chupakhin - chupakhin@ios.uran.ru

*Corresponding author

Experimental procedures, characterization data, copies of the ^{1}H, ^{13}C, ^{11}B NMR spectra and X-ray diffraction studies

TABLE OF CONTENTS

General procedures S2
General method for the synthesis of *2H*-imidazole-1-oxides **1c,d** S2
General method for the synthesis of C-modified carboranes **4a–d** S3
General method for the synthesis of C-modified carboranes **5a–d** S5
^{1}H, ^{11}B, and ^{13}C NMR Spectra S8
X-ray diffraction studies S42
References S43

General procedures

The ^{1}H NMR (400 MHz), ^{13}C NMR (100 MHz), ^{11}B (128 MHz) spectra were recorded using TMS, as the internal standard and $CDCl_3$ as a deuterated solvent. All $^{13}C\{^{1}H\}$ experiments were carried out in a mode of attached proton test (APT).The X-ray diffraction analysis was performed on a diffractometer, equipped with CDD detector (Mo KR graphite-monochromated radiation, λ = 0.71073 Å, ω-scanning technique, the scanning step was 1° and the exposure time per frame was 10 s at 295(2) K. Analytical absorption correction was used in the reflection intensity integration [1]. The structure was solved by the direct method and refined applying full matrix least-squares versus F^{2}_{hkl} with anisotropic displacement parameters for all non-hydrogen atoms using the SHELX97 program package [2]. All hydrogen atoms were located in different electron density maps and refined using a riding model with fixed thermal parameters. The mass spectra were recorded on a mass spectrometer with sample ionization by electron impact (EI). The IR spectra were recorded using a Fourier-transforminfrared spectrometer equipped with a diffuse reflection attachment. The elemental analysis was carried out on a CHNS/O analyzer. The course of the reactions was monitored by TLC on 0.25 mm silica gel plates (60F 254). Column chromatography was performed on silica gel (60, 0.040–0.063 mm (230–400 mesh)). *n*-BuLi (1.6 M solution in hexane), acetyl chloride, acetic anhydride, trifluoroacetic anhydride, 2,3-dichloro-5,6-dicyano-1,4-benzoquinone, 2,3,5,6-tetrachlorocyclohexa-2,5-diene-1,4-dione were purchased from Sigma-Aldrich. 2,2-Dimethyl-4-phenyl-2*H*-imidazole-1-oxide (**1a**) [3], 2-ethyl-2-methyl-4-phenyl-2*H*-imidazole-1-oxide (**1b**) [4] were prepared according to the published procedures.

General method for the synthesis of *2H*-imidazole-1-oxides 1c,d

A mixture of 2-(4-bromophenyl)-2-oxoacetaldehyde oxime (22 mmol), NH_4OAc (132 mmol), glacial acetic acid (264 mmol), and corresponding ketone (286 mmol) was heated under reflux for 2 h. The reaction mixture was then cooled, and water (1500 mL) was added. The organic phase was separated, and the aqueous phase was extracted with $CHCl_3$ (4 × 100 mL). The combined organic

layers were washed with 10 % NaOH, dried over Na_2SO_4, and concentrated under reduced pressure. The residue was purified by recrystallization from heptane.

4-(4-Bromophenyl)-2,2-dimethyl-*2H*-imidazole-1-oxide (1c). Yield 3.76 g (64%), mp 138-140 °C. ^{1}H NMR (400 MHz, $CDCl_3$, δ): 7.74 (d, J=8 Hz, 2H, Ar), 7.67 (s, 1H, HetAr), 7.64 (d, J=8 Hz, 2H, Ar), 1.62 (s, 6H, $C\underline{H}_3$). ^{13}C NMR (100 MHz, $CDCl_3$, δ): 163.98 (C), 132.48 (CH), 130.11 (C), 128.75 (CH), 126.70 (C), 124.92 (CH), 102.58 (C), 24.54 (CH_3). IR (DRA): 3083, 2990, 2932, 1592, 1553, 1503, 1484, 1409, 1357, 1219, 1196, 1064, 1004, 917, 816, 637 cm^{-1}. MS (EI): *m/z* 267 $[M]^+$. Anal. Calcd for $C_{11}H_{11}N_2OBr$: C, 49.46; H, 4.15; Br, 29.91; N, 10.49; O, 5.99. Found: C, 49.51; H, 4.19; N, 10.39.

4-(4-Bromophenyl)-2-ethyl-2-methyl-2*H*-imidazole-1-oxide (1d). Yield 3.585 g (58%), mp 108-110 °C. ^{1}H NMR (400 MHz, $CDCl_3$, δ):7.75 (d, J=8 Hz, 2H, Ar), 7.71 (s, 1H, HetAr), 7.64 (d, J=8 Hz, 2H, Ar), 2.24-2.10 (m, 1H, $C\underline{H}_2$), 2.08-1.95 (m, 1H, $C\underline{H}_2$), 1.61 (s, 3H, $C\underline{H}_3$), 0.67 (t, J=8 Hz, 3H, $C\underline{H}_3$),. ^{13}C NMR (100 MHz, $CDCl_3$, δ): 164.70 (C), 132.47 (CH), 130.05 (C), 128.72 (CH), 126.65 (C), 125.95 (CH), 105.18 (C), 30.77 (C H_2), 23.87 (CH_3), 7.09 (CH_3). IR (DRA): 3072, 2971, 2930, 1592, 1554, 1506, 1485, 1410, 1371, 1176, 1067, 1006, 921, 820, 659 cm^{-1}. MS (EI): *m/z* 293 $[M]^+$. Anal. Calcd for $C_{12}H_{13}N_2OBr$: C, 51.26; H, 4.66; Br, 28.42; N, 9.96; O, 5.69. Found: C, 51.32; H, 4.86; N, 9.79.

General method for the synthesis of C-modified carboranes 4a–d

In a similar manner to a procedure from [5], to a vigorously stirring solution of 1,2-*closo*-carborane (1.39 mmol, 200 mg) in dry THF at −78 °C under argon, a 1.6 M solution of *n*-BuLi in hexane (1.53 mmol, 0.955 mL) was added. The mixture was stirred at −78 °C for 30 min, then was warmed up to 0 °C and was stirred for additional 1 h. The mixture was cooled to –78 °C and solutions of the *2H*-imidazole-1-oxide **1** (1.53 mmol) was added. The resulting solution was allowed to warm up to ambient temperature and was stirred for 15 min. Then a solution of AcCl (1.53 mmol, 0.1 mL) was added and the resulted mixture was stirred for 2 h. The resulted mixture was subjected to silica gel

column chromatography with the EtOAc–hexane mixture as an eluent, the resulting eluate was concentrated to dryness under reduced pressure.

1-(2,2-Dimethyl-5-phenyl-2*H*-imidazol-4-yl)-1,2-dicarba-*closo*-dodecaborane (4a)

Yield 187 mg (44%), mp 126-128 °C, R_f 0.6 (hexane/EtOAc, 8:2). ^{1}H NMR (400 MHz, $CDCl_3$, δ): 7.53-7.40 (m, 3H, Ph), 7.39-7.33 (m, 2H, Ph), 4.58 (s, 1H, C(B)-H), 2.89-1.63 (m, 10H, B-H), 1.50 (s, 6H, C$\underline{H}_3$). ^{13}C NMR (100 MHz, $CDCl_3$, δ): 163.01 (C), 158.83 (C), 132.65 (C), 130.14 (CH), 128.41 (CH), 128.30 (CH), 100.77 (C), 69.32 (C(B)), 61.55 (C(B)-H), 23.38 (CH_3). ^{11}B NMR (128 MHz, $CDCl_3$, δ): 5.54-(-5.57) (m, 2B), -6.74-(-25.17) (m, 8B). IR (DRA): 3291, 3067, 2981, 2932, 2896, 2561, 1489, 1369, 1212, 1134, 1018, 906, 720, 696, 586 cm^{-1}. MS (EI): *m/z* 314 $[M]^+$. Anal. Calcd for $C_{13}H_{22}N_2B_{10}$: C, 49.66; H, 7.05; B, 34.38; N, 8.91. Found: C, 49.82; H, 6.86; N, 8.49.

1-(2-Ethyl-2-methyl-5-phenyl-2*H*-imidazol-4-yl)-1,2-dicarba-*closo*-dodecaborane (4b)

Yield 182 mg (40%), mp 106-108 °C, R_f 0.5 (hexane/EtOAc, 8:2). ^{1}H NMR (400 MHz, $CDCl_3$, δ): 7.53-7.40 (m, 3H, Ph), 7.38-7.33 (m, 2H, Ph), 4.58 (s, 1H, C(B)-H), 2.81-1.69 (m, 10H, B-H), 2.17-1.99 (m, 2H, C$\underline{H}_2$$CH_3$), 1.49 (s, 3H, C$\underline{H}_3$), 0.66 (t, J=7.36, 3H, CH_2C$\underline{H}_3$). ^{13}C NMR (100 MHz, $CDCl_3$, δ): 163.75 (C), 159.34 (C), 132.83 (C), 130.13 (CH), 128.44 (CH), 128.35 (CH), 103.07 (C), 69.36 (C(B)), 61.47 (C(B)-H), 30. 48 (CH_2), 22.24 (CH_3), 7.99 (CH_3). ^{11}B NMR (128 MHz, $CDCl_3$, δ): 3.19-(-5.53) (m, 2B), -5.98-(-28.78) (m, 8B). IR (DRA): 3217, 3026, 2975, 2925, 2635, 2576, 1552, 1445, 1262, 1176, 1014, 908, 801, 757, 721, 695, 613 cm^{-1}. MS (EI): *m/z* 328 $[M]^+$. Anal. Calcd for $C_{14}H_{24}N_2B_{10}$: C, 51.19; H, 7.36; B, 32.91; N, 8.53. Found: C, 50.85; H, 7.37; N, 8.11.

1-(5-(4-Bromophenyl)-2,2-dimethyl-2*H*-imidazol-4-yl)-1,2-dicarba-*closo*-dodecaborane (4c)

Yield 300 mg (55%), mp 102-104 °C, R_f 0.3 (hexane/EtOAc, 9:1). ^{1}H NMR (400 MHz, $CDCl_3$, δ): 7.62-7.56 (m, 2H, Ph), 7.29-7.23 (m, 2H, Ph), 4.63 (s, 1H, C(B)-H), 2.91-1.63 (m, 10H, B-H), 1.49 (s, 6H, C$\underline{H}_3$). ^{13}C NMR (100 MHz, $CDCl_3$, δ): 162.14 (C), 158.59 (C), 131.67 (CH), 131.64 (C), 130.05 (CH), 124.67 (C), 101.01 (C), 69.12 (C(B)), 61.75 (C(B)-H), 23.33 (CH_3). ^{11}B NMR (128 MHz, $CDCl_3$, δ): 4.67-(-5.33) (m, 2B), -6.03-(-28.14) (m, 8B). IR (DRA): 3211, 3070, 2988, 2934, 2850, 2576, 1726, 1591, 1481, 1392, 1263, 1176, 1070, 1012, 911, 823, 717, 636 cm^{-1}. MS (EI): *m/z* 393

$[M]^+$. Anal. Calcd for $C_{13}H_{21}N_2BrB_{10}$: C, 39.70; H, 5.38; B, 27.49; Br, 20.31; N, 7.12. Found: C, 39.47; H, 5.17; N, 6.79; Br, 19.98.

1-(5-(4-Bromophenyl)-2-ethyl-2-methyl-2*H*-imidazol-4-yl)-1,2-dicarba-*closo*-dodecaborane (4d)

Yield 277 mg (49%), mp 84-86 °C, R_f 0.3 (hexane/EtOAc, 9:1).^{1}H NMR (400 MHz, $CDCl_3$, δ): 7.63-7.55 (m, 2H, Ph), 7.29-7.22 (m, 2H, Ph), 4.63 (s, 1H, C(B)-H), 2.91-1.61 (m, 10H, B-H), 2.13-1.99 (m, 2H, C$\underline{H_2}$$CH_3$), 1.48 (s, 3H, C$\underline{H_3}$), 0.65 (t, J=7.36, 3H, CH_2C$\underline{H_3}$). ^{13}C NMR (100 MHz, $CDCl_3$, δ): 162.83 (C), 159.08 (C), 131.79 (C), 131.69 (CH), 130.08 (CH), 124.63 (C), 103.31 (C), 69.15 (C(B)), 61.67 (C(B)-H), 30.48 (CH_2), 22.16 (CH_3), 8.05 (CH_3). ^{11}B NMR (128 MHz, $CDCl_3$, δ): 3.27-(-26.46) (m, 10B). IR (DRA): 3217, 3069, 2971, 2926, 2853, 2605, 2577, 1621, 1593, 1483, 1466, 1392, 1286, 1172, 1070, 1012, 966, 827, 715 cm^{-1}. MS (EI): *m/z* 407 $[M]^+$. Anal. Calcd for $C_{14}H_{23}N_2BrB_{10}$: C, 41.28; H, 5.69; B, 26.54; Br, 19.62; N, 6.88. Found: C, 41.12; H, 5.26; N, 6.49.

General method for the synthesis of C-modified carboranes 5a–d

To a vigorously stirring solution of 1,2-*closo*-carborane (1.39 mmol, 200 mg) in dry THF at -78 °C under argon, a 1.6 M solution of *n*-BuLi in hexane (1.53 mmol, 0.955 mL) was added. The mixture was stirred at -78 °C for 30 min, then was warmed up to 0 °C and was stirred for additional 1 h. The mixture was cooled to –78 °C and solutions of the *2H*-imidazole-1-oxide **1** (1.53 mmol) was added. The resulting solution was allowed to warm up to ambient temperature and was stirred for 15 min. Then a solution of DDQ in THF (1.53 mmol) was added and the resulted mixture was refluxed for 1 h under argon. The resulted mixture was subjected to aluminia column chromatography with the EtOAc as an eluent, the resulting eluate was concentrated to dryness in vacuo and the residue was subjected to silica gel column chromatography with the EtOAc–hexane mixture as an eluent, the resulting eluate was concentrated to dryness under reduced pressure.

1-(2,2-Dimethyl-1-oxido-4-phenyl-2*H*-imidazol-5-yl)-1,2-dicarba-*closo*-dodecaborane (5a)

Yield 192 mg (42%), mp 112-114 °C, R_f 0.4 (hexane/EtOAc, 1:1).^{1}H NMR (400 MHz, $CDCl_3$, δ): 7.56-7.44 (m, 3H, Ph), 7.42-7.36 (m, 2H, Ph), 6.03 (s, 1H, C(B)-H), 3.08-1.65 (m, 10H, B-H), 1.59 (s,

6H, C$\underline{H}_3$). ^{13}C NMR (100 MHz, $CDCl_3$, δ): 166.41 (C), 133.08 (C), 130.54 (CH), 129.18 (C), 128.53 (CH), 128.35 (C), 101.38 (C), 65.66 (C(B)), 57.64 (C(B)-H), 24.71 (CH_3). ^{11}B NMR (128 MHz, $CDCl_3$, δ): 20.83-(-5.15) (m, 3B), -6.76-(-29.08) (m, 7B). IR (DRA): 3061, 2997, 2985, 2935, 2653, 2576, 1612, 1505, 1366, 1247, 1059, 958, 755, 721, 692, 656 cm^{-1}. MS (EI): *m/z* 330 $[M]^+$. Anal. Calcd for $C_{13}H_{22}N_2OB_{10}$: C, 47.25; H, 6.71; B, 32.72; N, 8.48; O, 4.84. Found: C, 47.42; H, 6.76; N, 8.00.

1-(2-Ethyl-2-methyl-1-oxido-4-phenyl-2*H*-imidazol-5-yl)-1,2-dicarba-*closo*-dodecaborane (5b)

Yield 186 mg (39%), mp 138-140 °C, R_f 0.4 (hexane/EtOAc, 8:2).^{1}H NMR (400 MHz, $CDCl_3$, δ): 7.56-7.44 (m, 3H, Ph), 7.42-7.35 (m, 2H, Ph), 6.06 (s, 1H, C(B)-H), 2.92-1.64 (m, 10H, B-H), 2.23-2.10 (m, 1H, C$\underline{H_2}$CH$_3$), 2.08-1.98 (m, 1H, C$\underline{H_2}$CH$_3$), 1.59 (s, 3H, C$\underline{H_3}$), 0.62 (t, J=7.32, 3H, CH$_2$C$\underline{H_3}$). ^{13}C NMR (100 MHz, $CDCl_3$, δ): 167.38 (C), 133.23 (C), 130.59 (C), 130.52 (CH), 128.54 (CH), 128.39 (CH), 103.64 (C), 65.56 (C(B)), 57.63 (C(B)-H), 31.09(CH_2), 24.10 (CH_3), 6.71(CH_3). ^{11}B NMR (128 MHz, $CDCl_3$, δ): 15.54-(-6.22) (m, 2B), -7.20-(-32.40) (m, 8B). IR (DRA): 3208, 3048, 2994, 2922, 2595, 2571, 1576, 1487, 1388, 1317, 1062, 961, 751, 720, 691 cm^{-1}. MS (EI): *m/z* 344 $[M]^+$. Anal. Calcd for $C_{14}H_{24}N_2OB_{10}$: C, 48.82; H, 7.02; B, 31.39; N, 8.13; O, 4.64. Found: C, 48.54; H, 7.12; N, 7.83.

1-(4-(4-Bromophenyl)-2,2-dimethyl-1-oxido-2*H*-imidazol-5-yl)-1,2-dicarba-*closo*-dodecaborane (5c)

Yield 296 mg (52%), mp 136-138 °C, R_f 0.2 (hexane/EtOAc, 9:1).^{1}H NMR (400 MHz, $CDCl_3$, δ): 7.66-7.59 (m, 2H, Ph), 7.31-7.26 (m, 2H, Ph), 6.06 (s, 1H, C(B)-H), 2.83-1.66 (m, 10H, B-H), 1.57 (s, 6H, C$\underline{H}_3$). ^{13}C NMR (100 MHz, $CDCl_3$, δ): 165.56 (C), 132.05 (C), 131.82 (CH), 130.05 (CH), 129.02 (C), 125.12 (C), 101.57 (C), 65.50 (C(B)), 57.64 (C(B)-H), 24.69 (CH_3). ^{11}B NMR (128 MHz, $CDCl_3$, δ): 8.03-(-5.44) (m, 2B), -6.36-(-31.70) (m, 8B). IR (DRA): 3220, 3075, 2988, 2933, 2854, 2598, 2561, 1601, 1504, 1362, 1313, 1243, 1173, 1069, 1012, 956, 832, 716, 647, 621 cm^{-1}. MS (EI): *m/z* 409 $[M]^+$. Anal. Calcd for $C_{13}H_{21}N_2OBrB_{10}$: C, 38.14; H, 5.17; B, 26.41; Br, 19.52; N, 6.84; O, 3.91. Found: C, 38.00; H, 5.35; N, 6.36; Br, 19.17.

1-(4-(4-Bromophenyl)-2-ethyl-2-methyl-1-oxido-2*H*-imidazol-5-yl)-1,2-dicarba-*closo*-dodecaborane (5d)

Yield 270 mg (46%), mp 154-156 °C, R_f 0.2 (hexane/EtOAc, 9:1). ^{1}H NMR (400 MHz, $CDCl_3$, δ): 7.67-7.58 (m, 2H, Ph), 7.31-7.26 (m, 2H, Ph), 6.09 (s, 1H, C(B)-H), 3.02-1.64 (m, 10H, B-H), 2.22-2.10 (m, 1H, $C\underline{H_2}CH_3$), 2.08-1.96 (m, 1H, $C\underline{H_2}CH_3$), 1.57 (s, 3H, $C\underline{H_3}$), 0.61 (t, J=7.32, 3H, $CH_2C\underline{H_3}$). ^{13}C NMR (100 MHz, $CDCl_3$, δ): 166.51 (C), 132.19 (C), 131.83 (CH), 130.41 (C), 130.08 (CH), 125.07 (C), 103.85 (C), 65.41 (C(B)), 57.64 (C(B)-H), 31.12(CH_2), 24.06 (CH_3), 6.74 (CH_3). ^{11}B NMR (128 MHz, $CDCl_3$, δ): 3.79-(-5.48) (m, 2B), -6.28-(-26.43) (m, 8B). IR (DRA): 3069, 2973, 2928, 2634, 2581, 1604, 1507, 1363, 1315, 1284, 1175, 1068, 1011, 961, 829, 713, 668, 534 cm^{-1}. MS (EI): *m/z* 423 $[M]^+$. Anal. Calcd for $C_{14}H_{23}N_2OBrB_{10}$: C, 39.72; H, 5.48; B, 25.54; Br, 18.87; N, 6.62; O, 3.78. Found: C, 40.01; H, 5.85; N, 6.36; Br, 18.54.

^{1}H, ^{11}B, and ^{13}C NMR Spectra

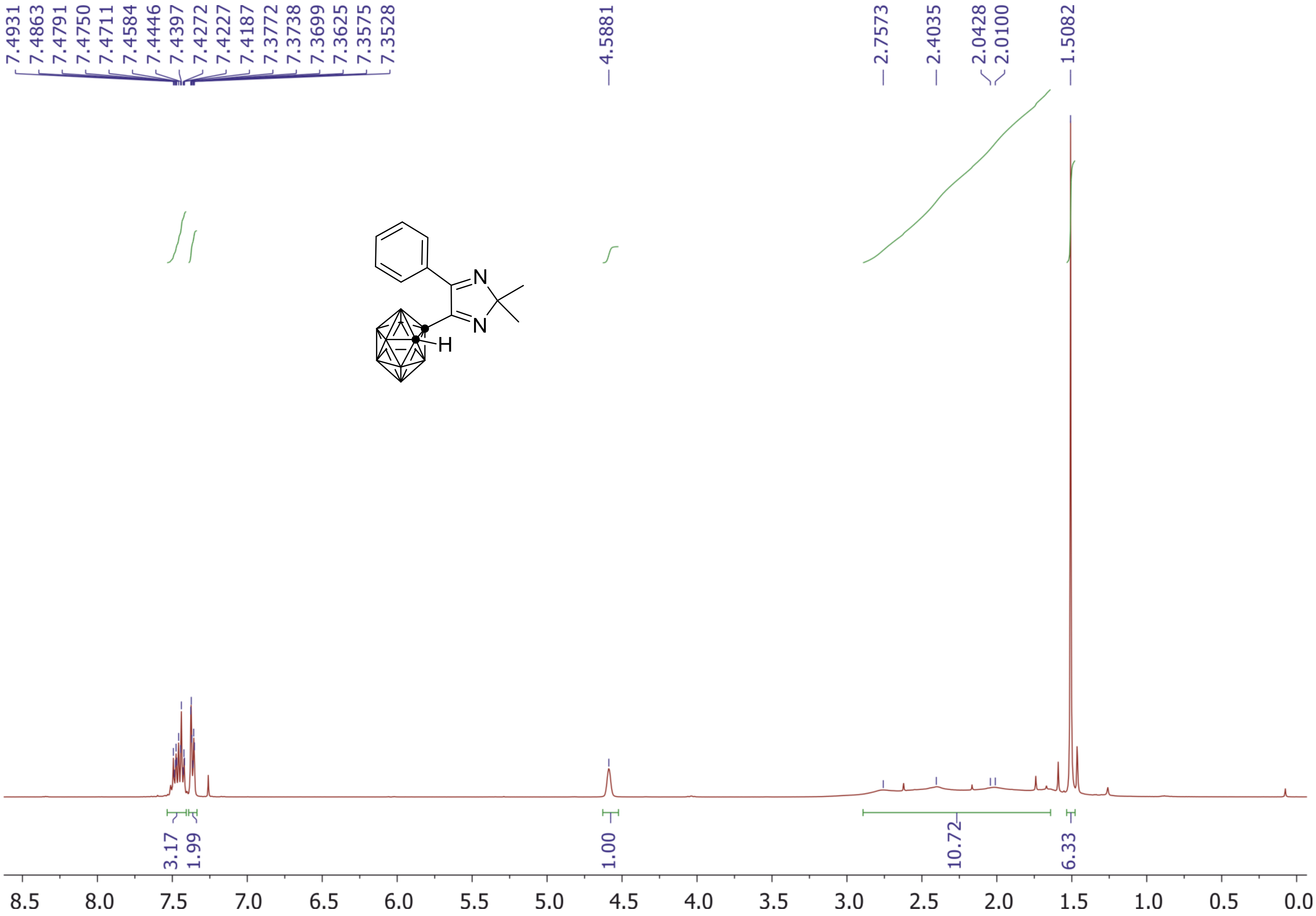

Figure S1. ^{1}H NMR Spectrum of 1-(2,2-dimethyl-5-phenyl-2*H*-imidazol-4-yl)-1,2-dicarba-*closo*-dodecaborane (**4a**).

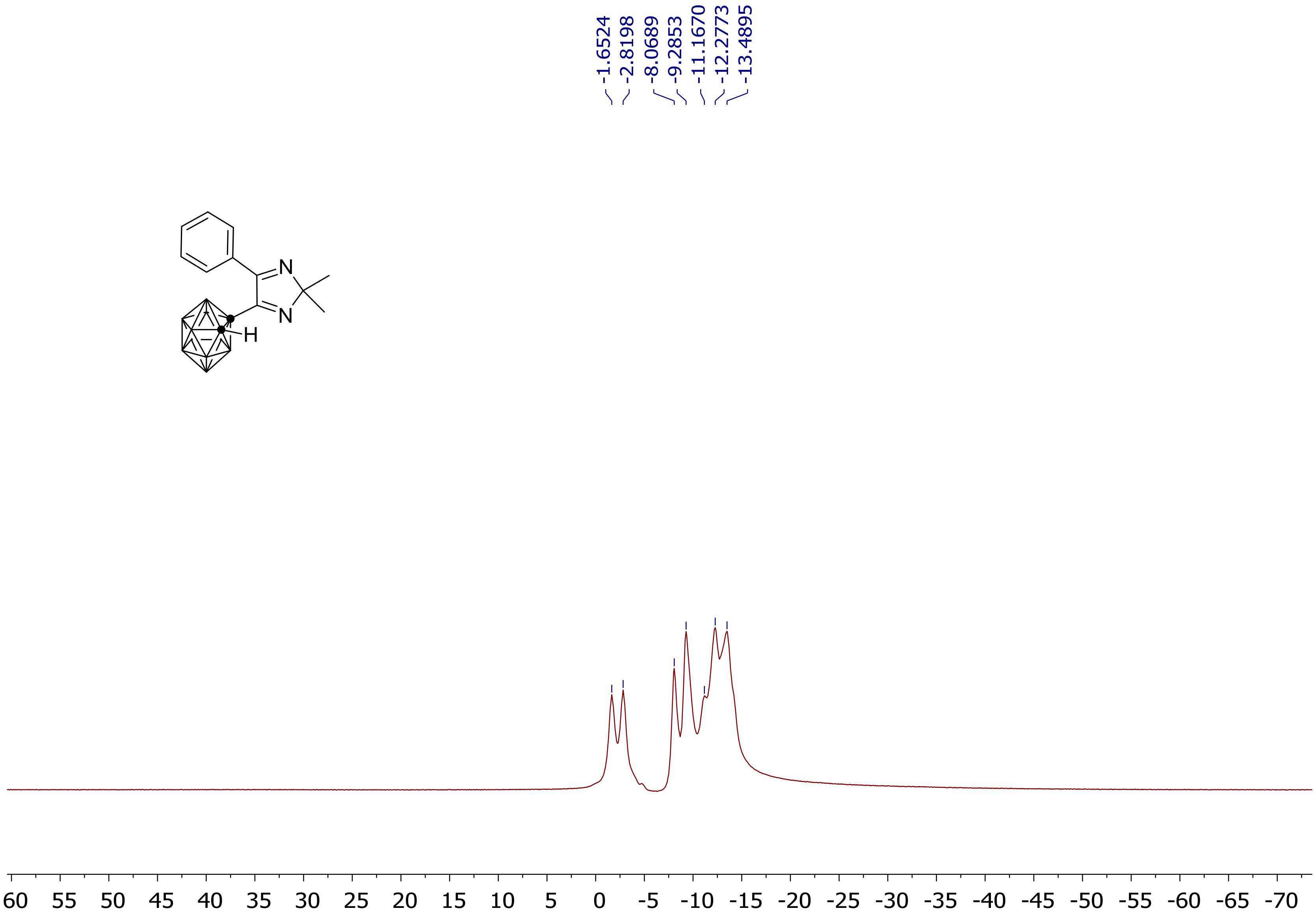

Figure S2. ^{11}B NMR Spectrum of 1-(2,2-dimethyl-5-phenyl-2*H*-imidazol-4-yl)-1,2-dicarba-*closo*-dodecaborane (**4a**).

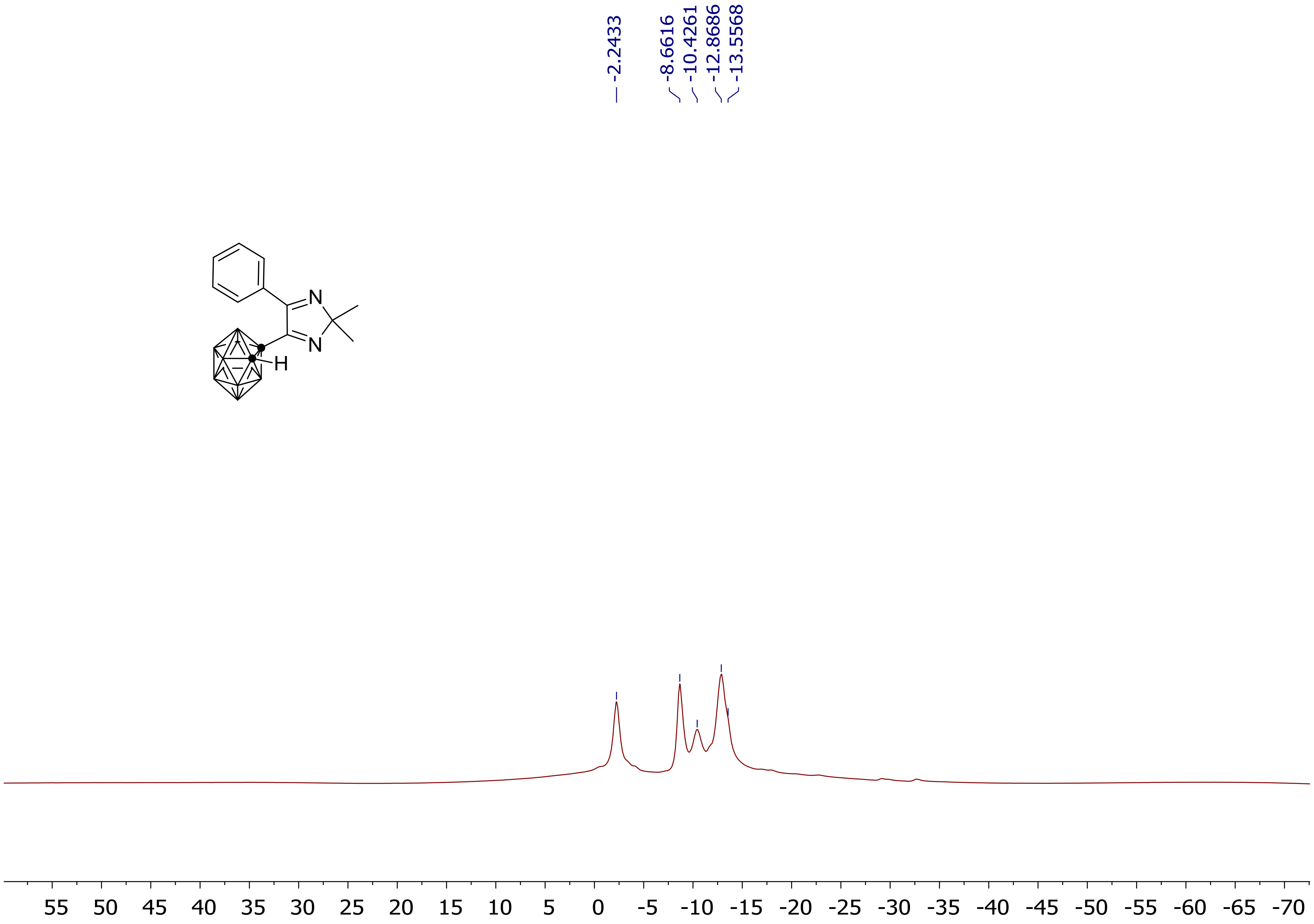

Figure S3. $^{11}B\{^{1}H\}$ NMR Spectrum of 1-(2,2-dimethyl-5-phenyl-2H-imidazol-4-yl)-1,2-dicarba-closo-dodecaborane (**4a**).

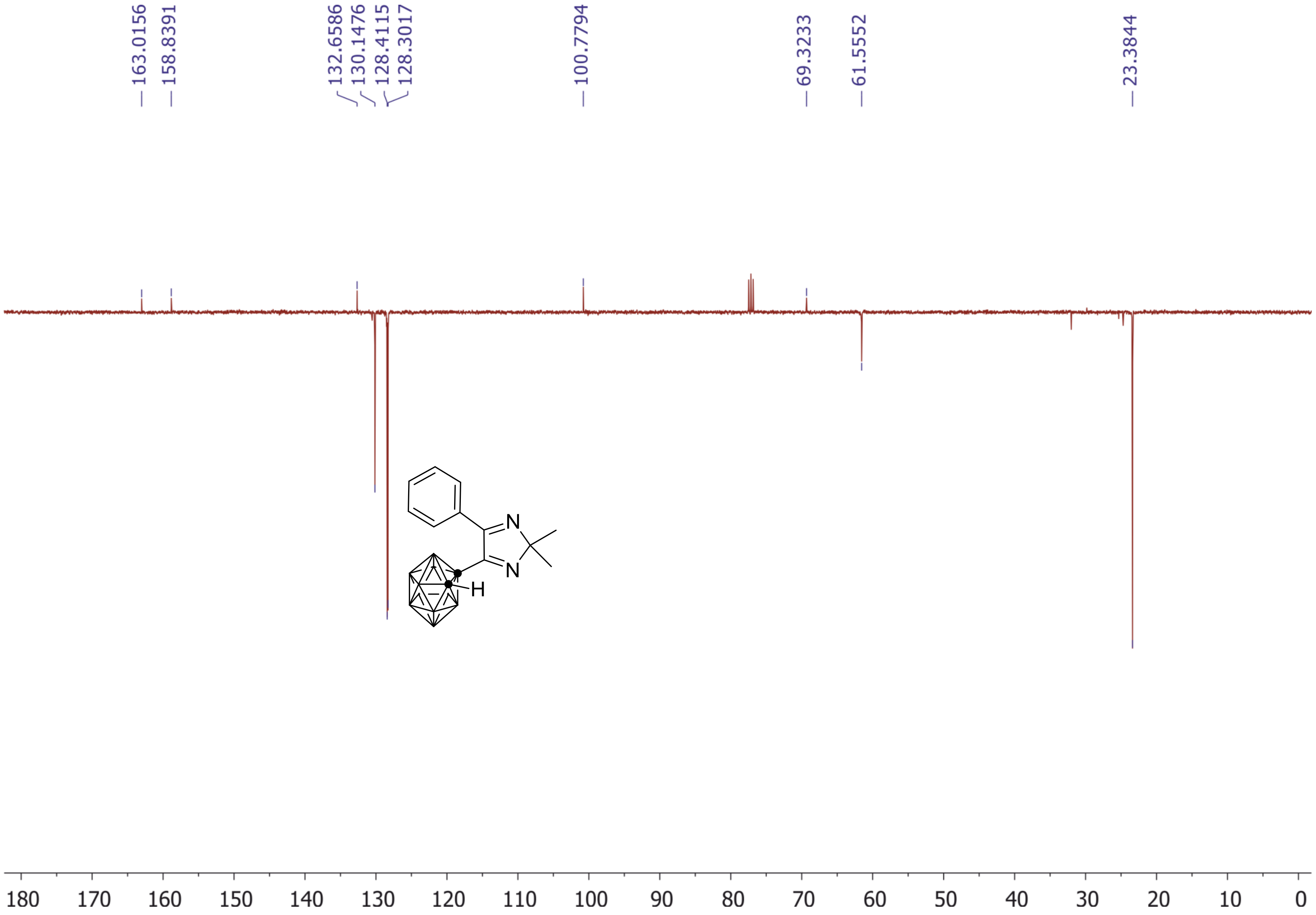

Figure S4. $^{13}C\{^{1}H\}$ NMR Spectrum (APT) of 1-(2,2-dimethyl-5-phenyl-2*H*-imidazol-4-yl)-1,2-dicarba-*closo*-dodecaborane (**4a**).

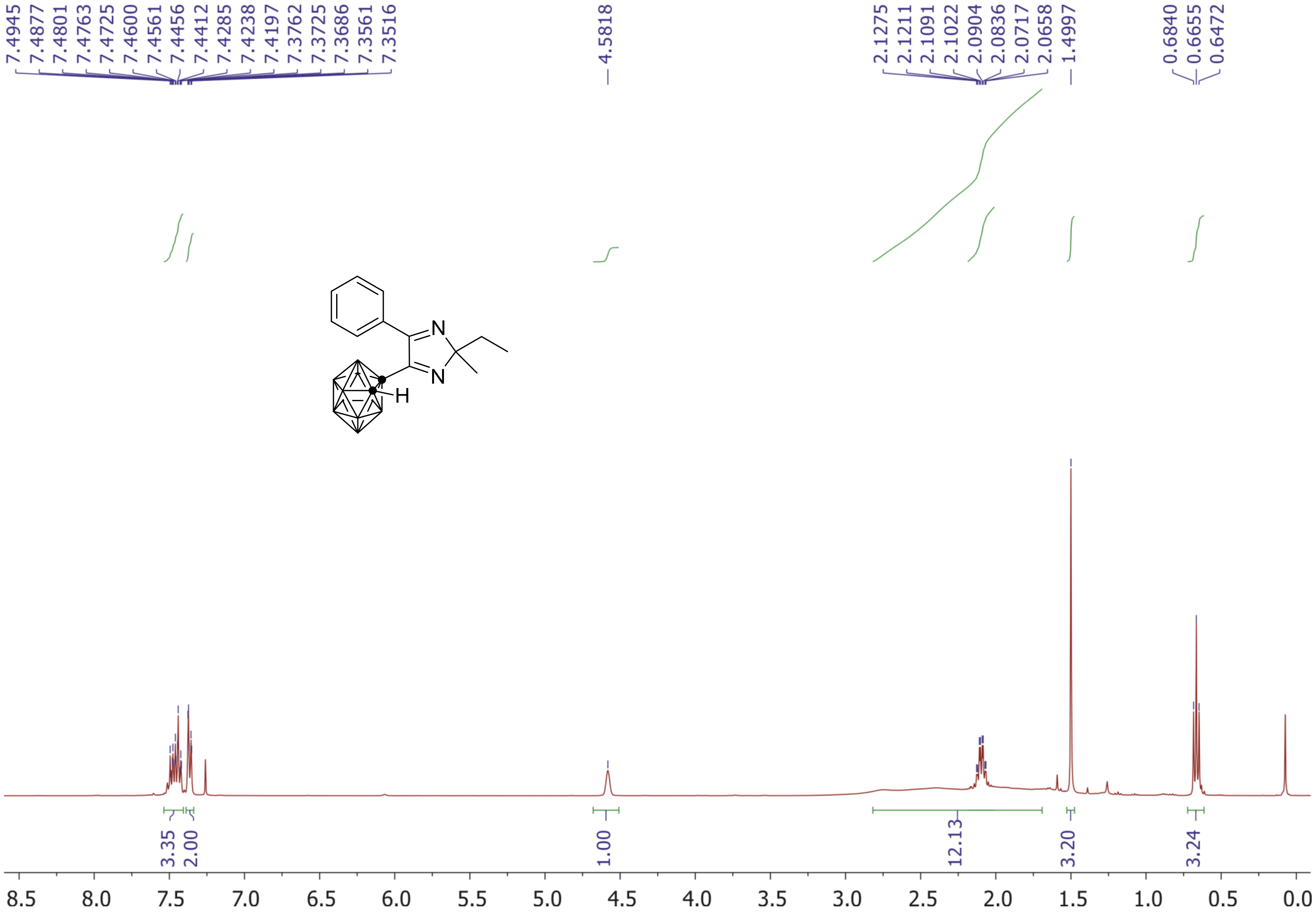

Figure S5. ^{1}H NMR Spectrum of 1-(2-ethyl-2-methyl-5-phenyl-2*H*-imidazol-4-yl)-1,2-dicarba-*closo*-dodecaborane (**4b**).

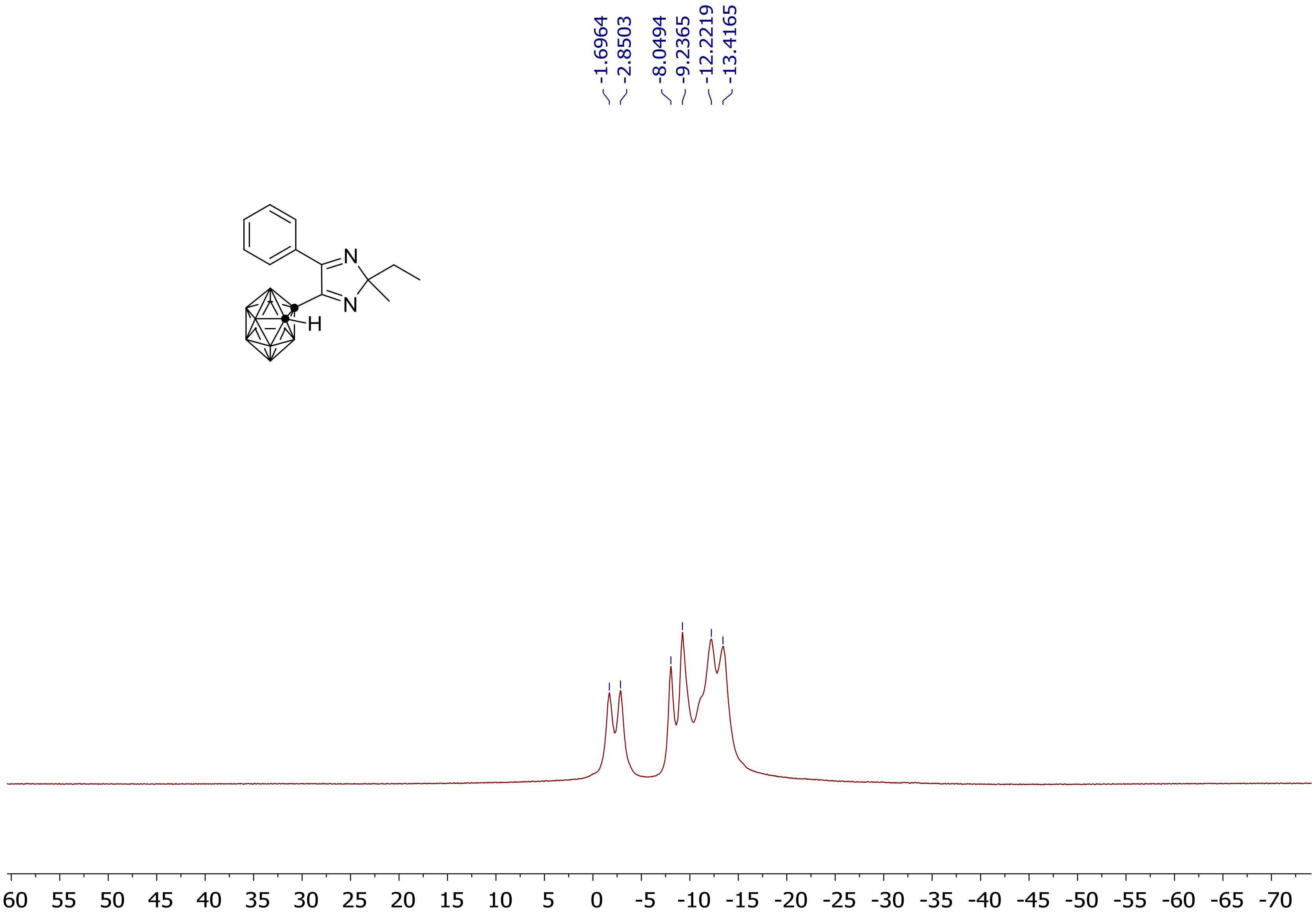

Figure S6. ^{11}B NMR Spectrum of 1-(2-ethyl-2-methyl-5-phenyl-2*H*-imidazol-4-yl)-1,2-dicarba-*closo*-dodecaborane (**4b**).

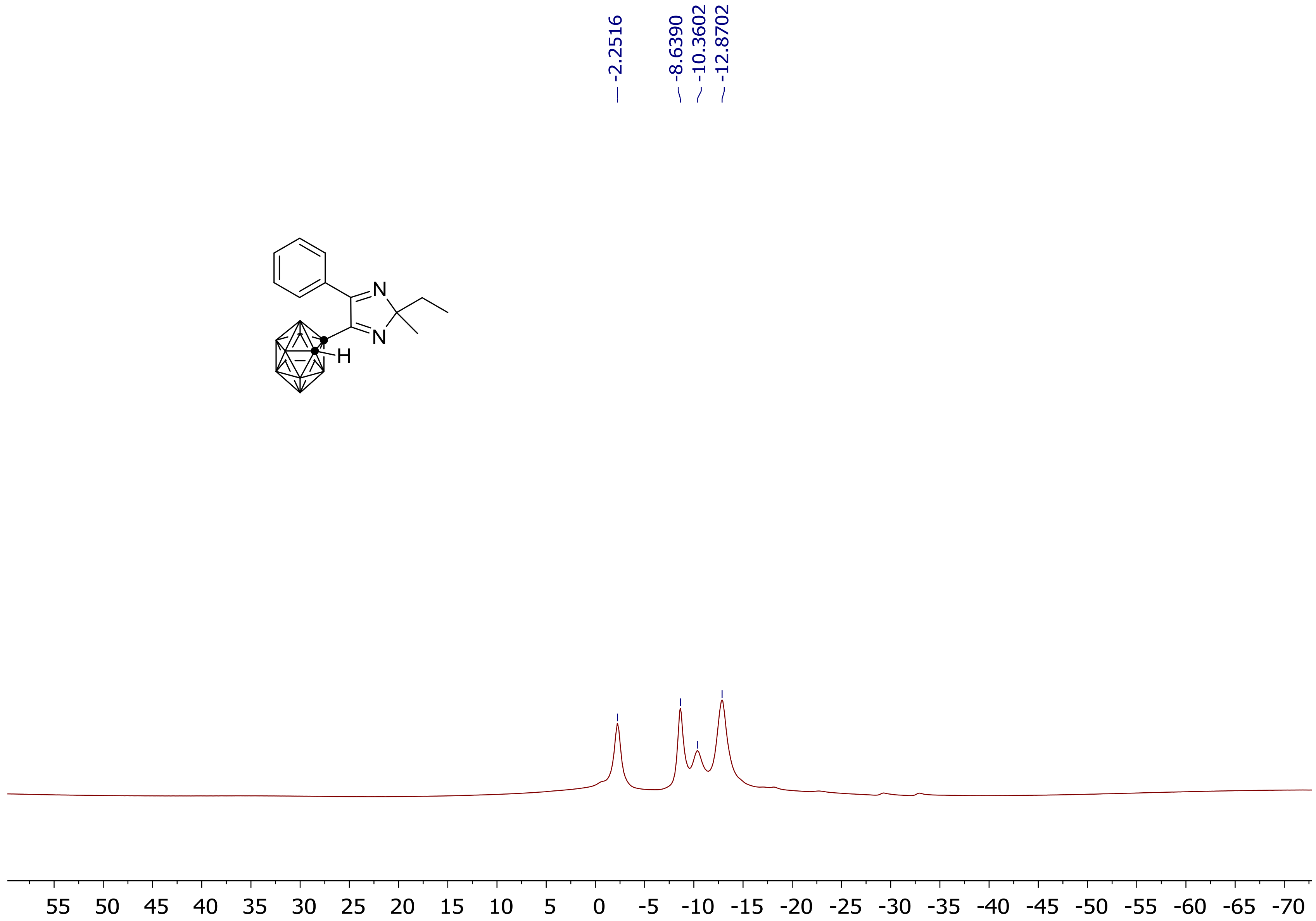

Figure S7. $^{11}B\{^{1}H\}$ NMR Spectrum of 1-(2-ethyl-2-methyl-5-phenyl-2*H*-imidazol-4-yl)-1,2-dicarba-*closo*-dodecaborane (**4b**).

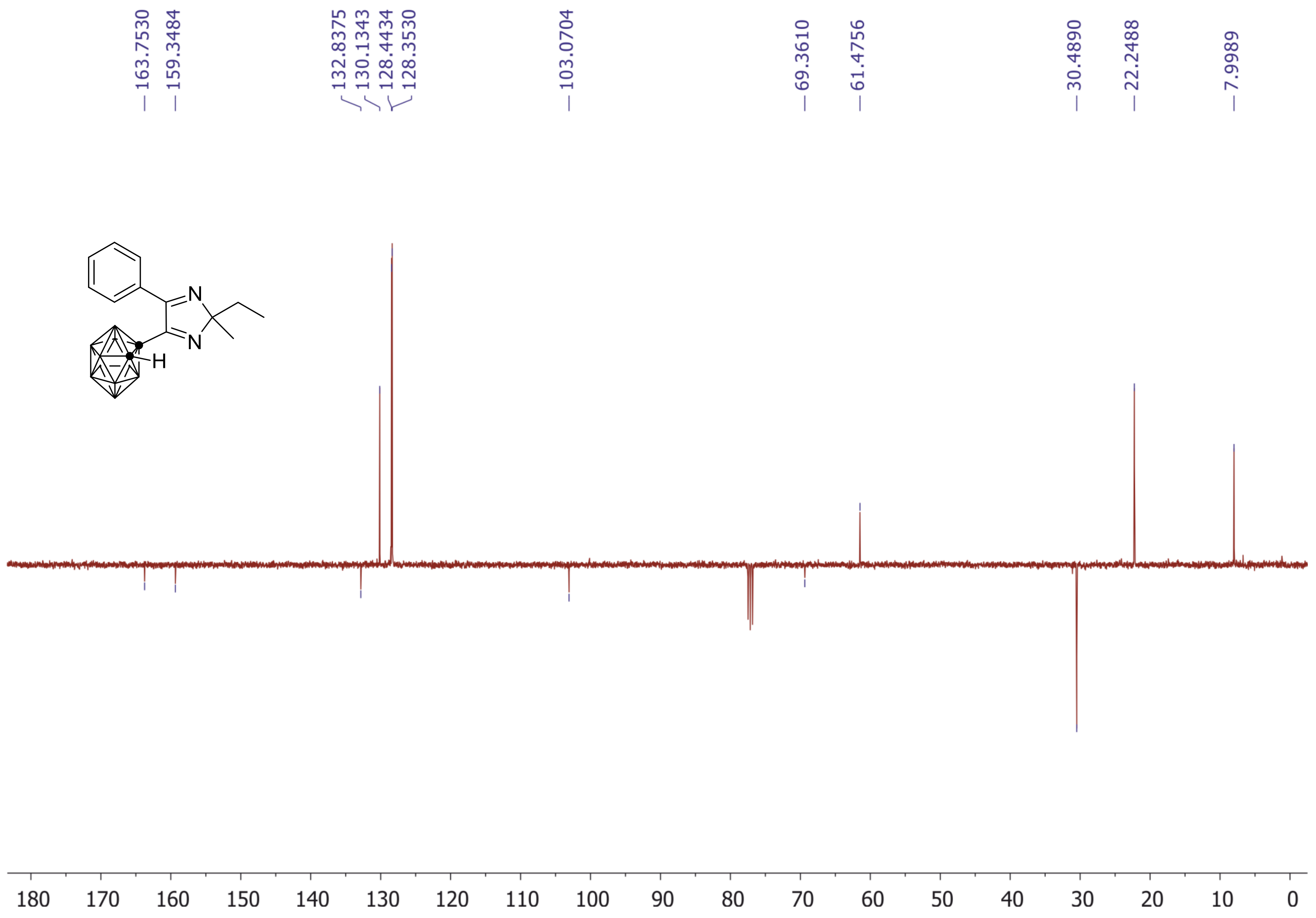

Figure S8. $^{13}C\{^{1}H\}$ NMR Spectrum (APT) of 1-(2-ethyl-2-methyl-5-phenyl-2*H*-imidazol-4-yl)-1,2-dicarba-*closo*-dodecaborane (**4b**).

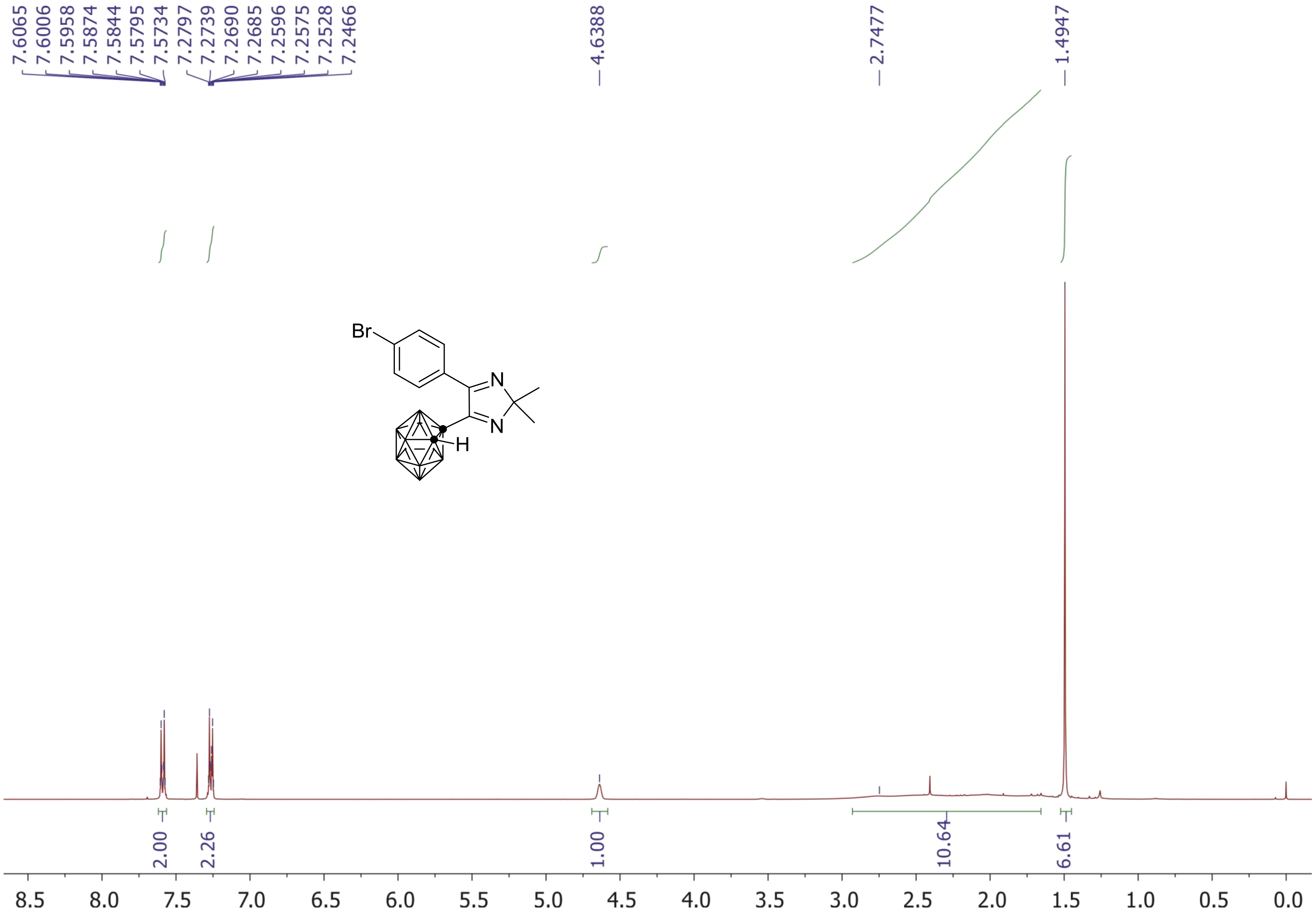

Figure S9. ^{1}H NMR Spectrum of 1-(5-(4-bromophenyl)-2,2-dimethyl-2*H*-imidazol-4-yl)-1,2-dicarba-*closo*-dodecaborane (**4c**).

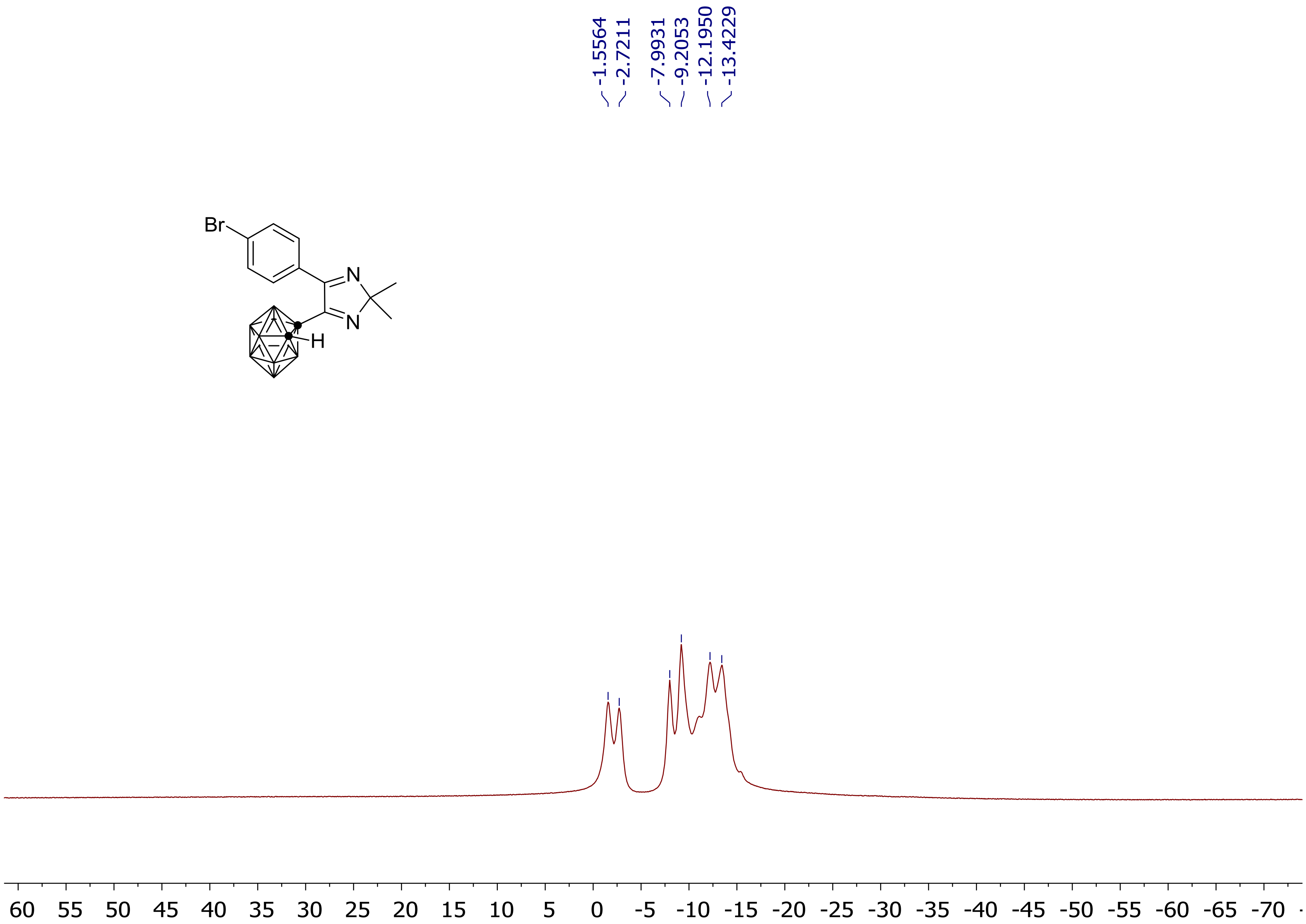

Figure S10. ^{11}B NMR Spectrum of 1-(5-(4-bromophenyl)-2,2-dimethyl-2*H*-imidazol-4-yl)-1,2-dicarba-*closo*-dodecaborane (**4c**).

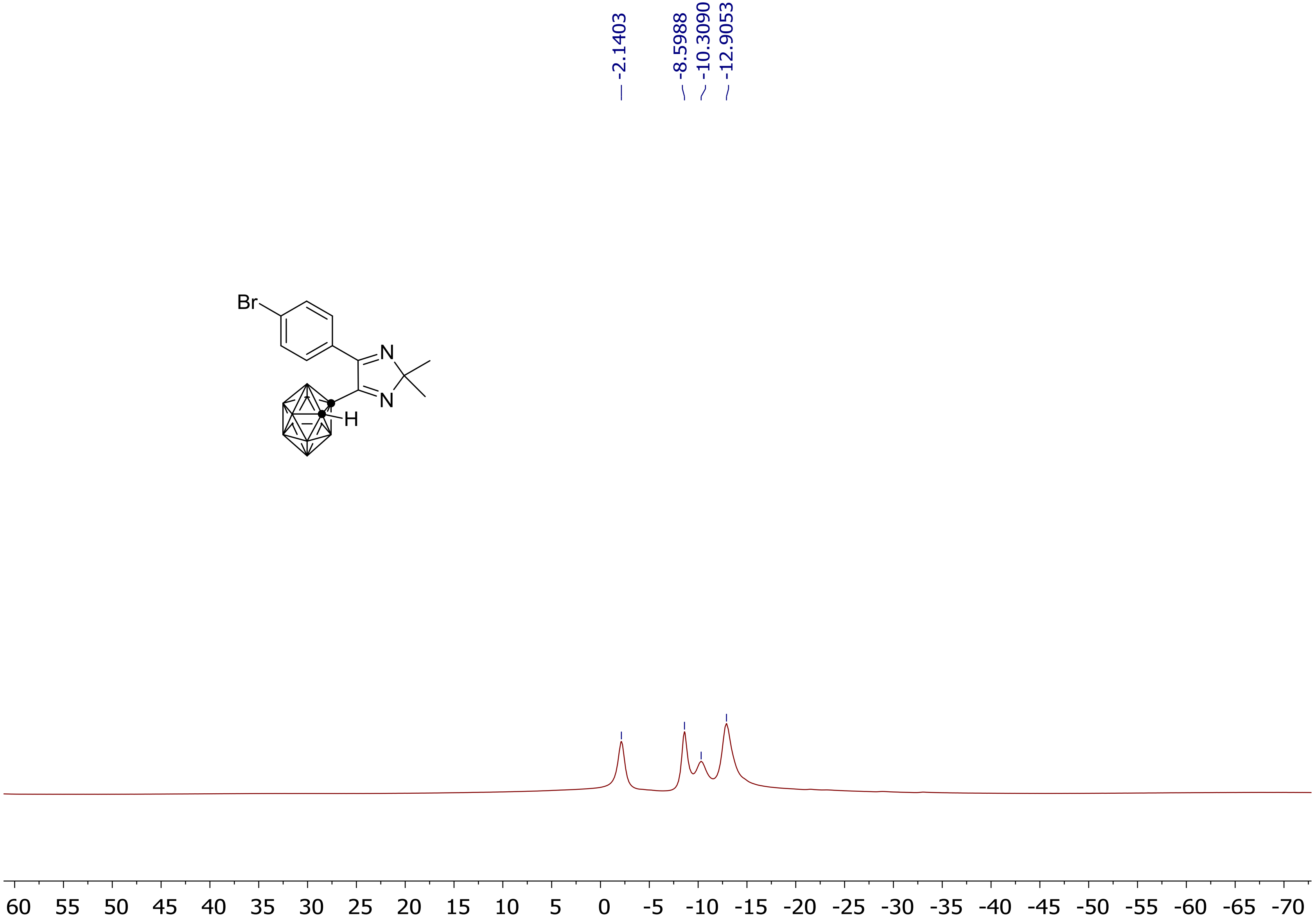

Figure S11. $^{11}B\{^{1}H\}$ NMR Spectrum of 1-(5-(4-bromophenyl)-2,2-dimethyl-2*H*-imidazol-4-yl)-1,2-dicarba-*closo*-dodecaborane (**4c**).

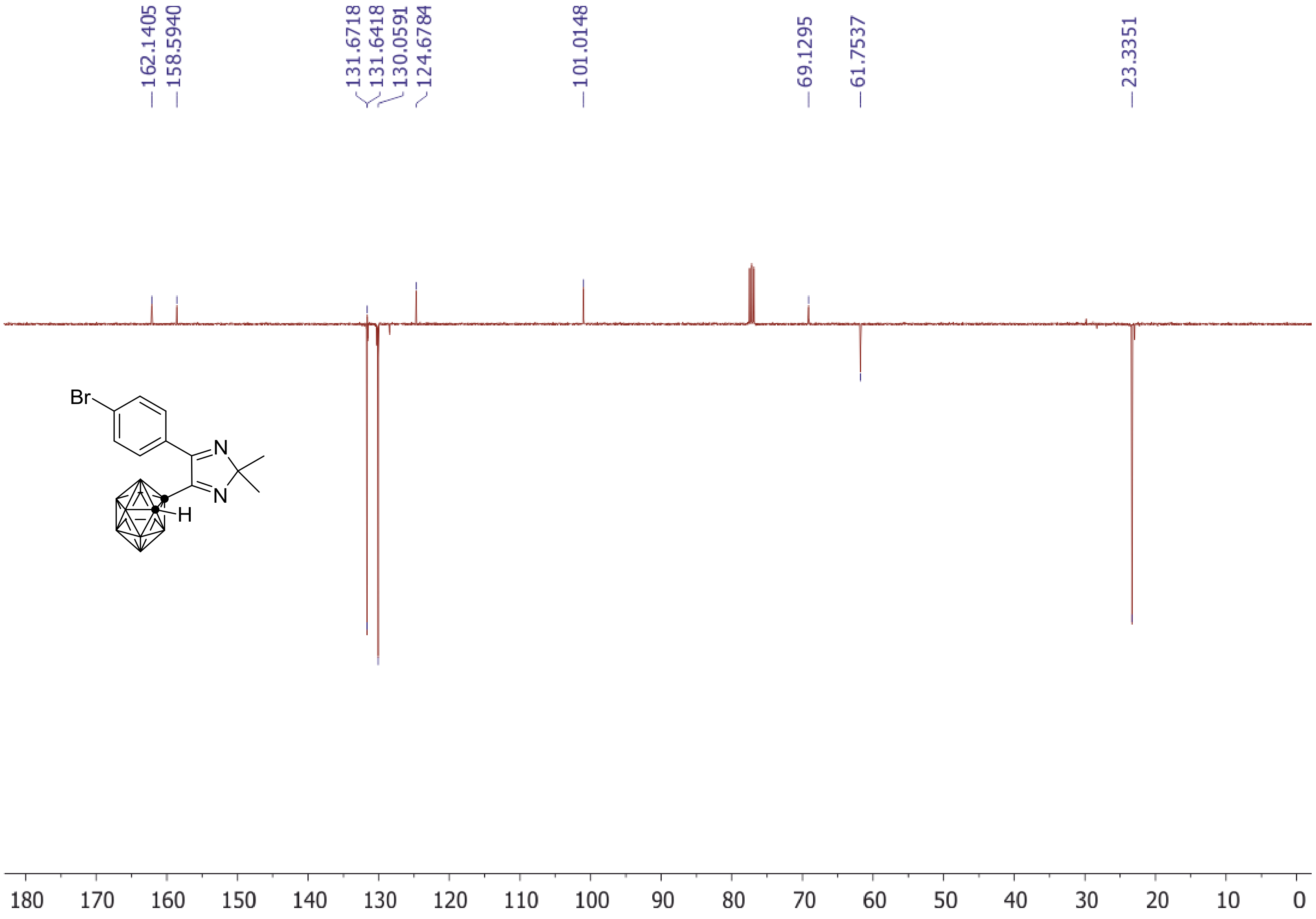

Figure S12. $^{13}C\{^{1}H\}$ NMR Spectrum (APT) of 1-(5-(4-bromophenyl)-2,2-dimethyl-2*H*-imidazol-4-yl)-1,2-dicarba-*closo*-dodecaborane (**4c**).

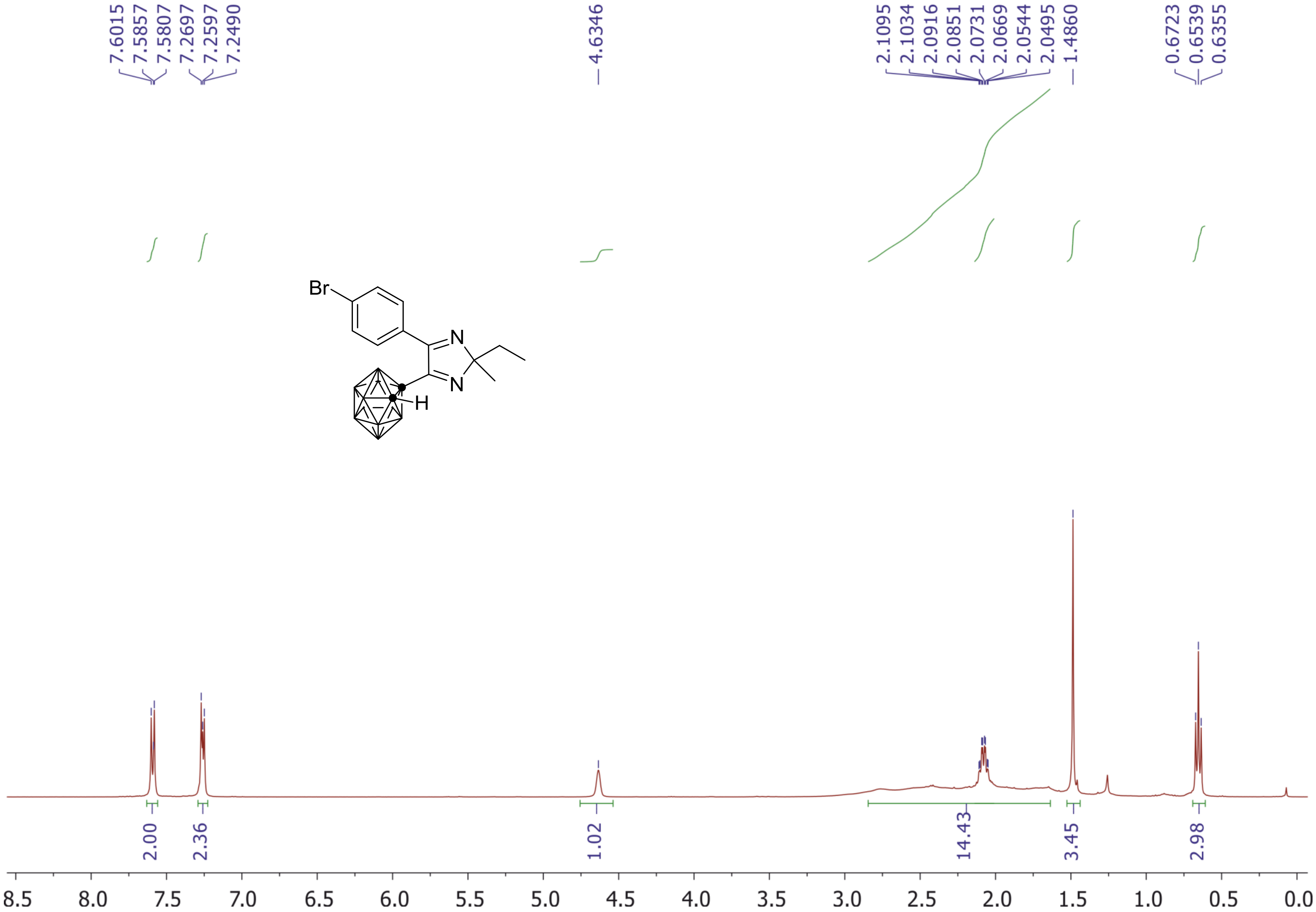

Figure S13. ^{1}H NMR Spectrum of 1-(5-(4-bromophenyl)-2-ethyl-2-methyl-2*H*-imidazol-4-yl)-1,2-dicarba-*closo*-dodecaborane (**4d**).

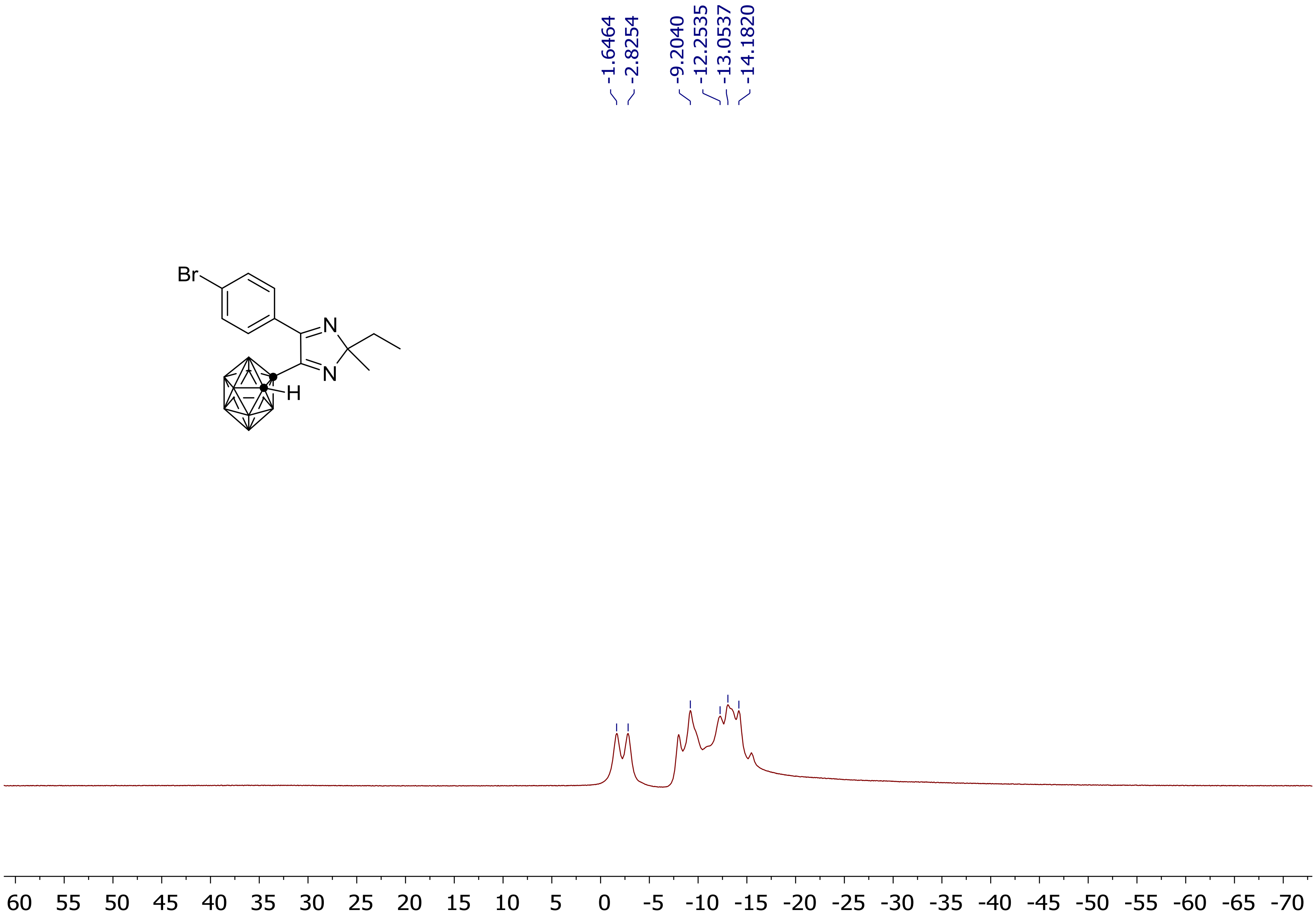

Figure S14. ^{11}B NMR Spectrum of 1-(5-(4-bromophenyl)-2-ethyl-2-methyl-2*H*-imidazol-4-yl)-1,2-dicarba-*closo*-dodecaborane (**4d**).

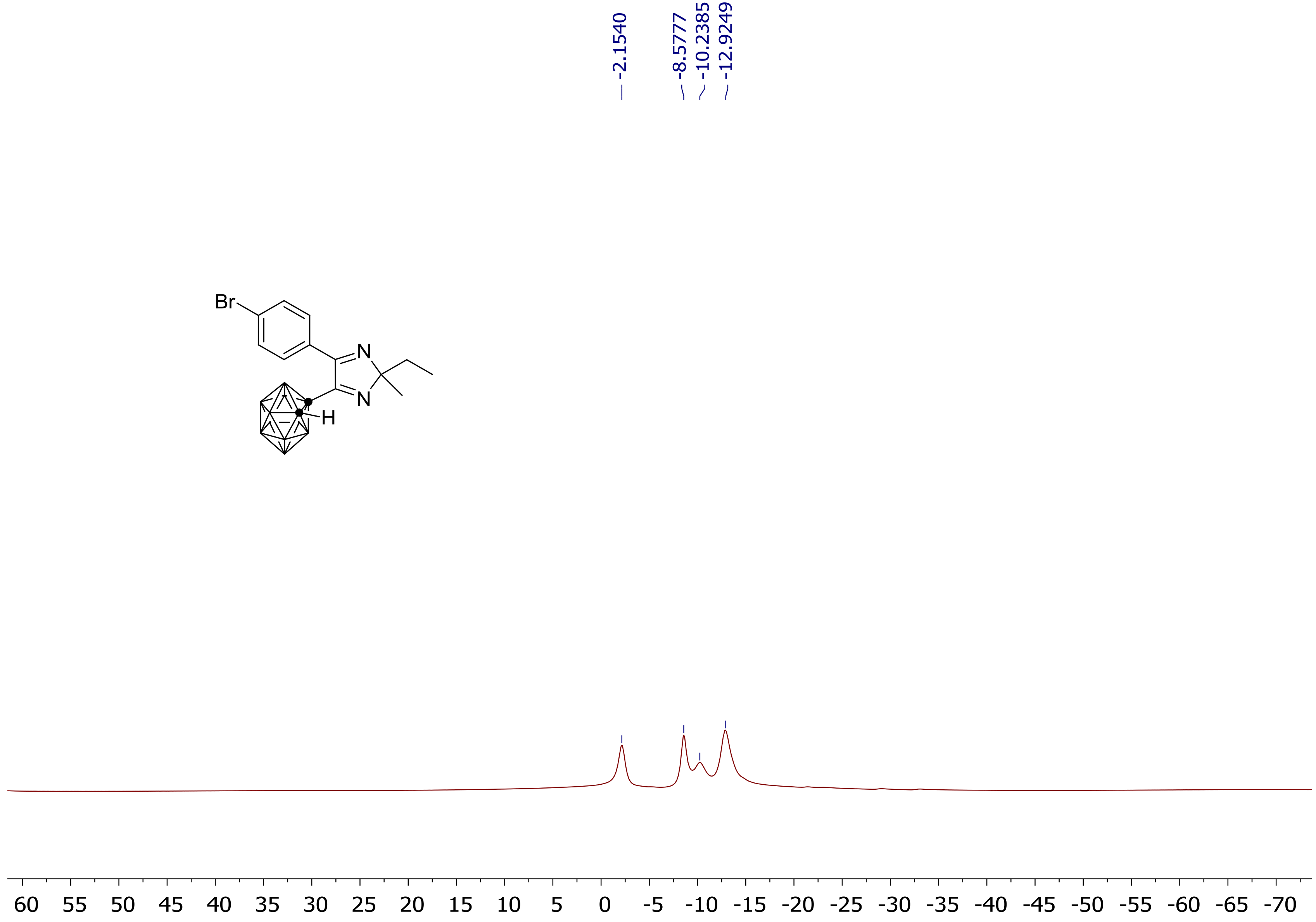

Figure S15. $^{11}B\{^1H\}$ NMR Spectrum of 1-(5-(4-bromophenyl)-2-ethyl-2-methyl-2*H*-imidazol-4-yl)-1,2-dicarba-*closo*-dodecaborane (**4d**).

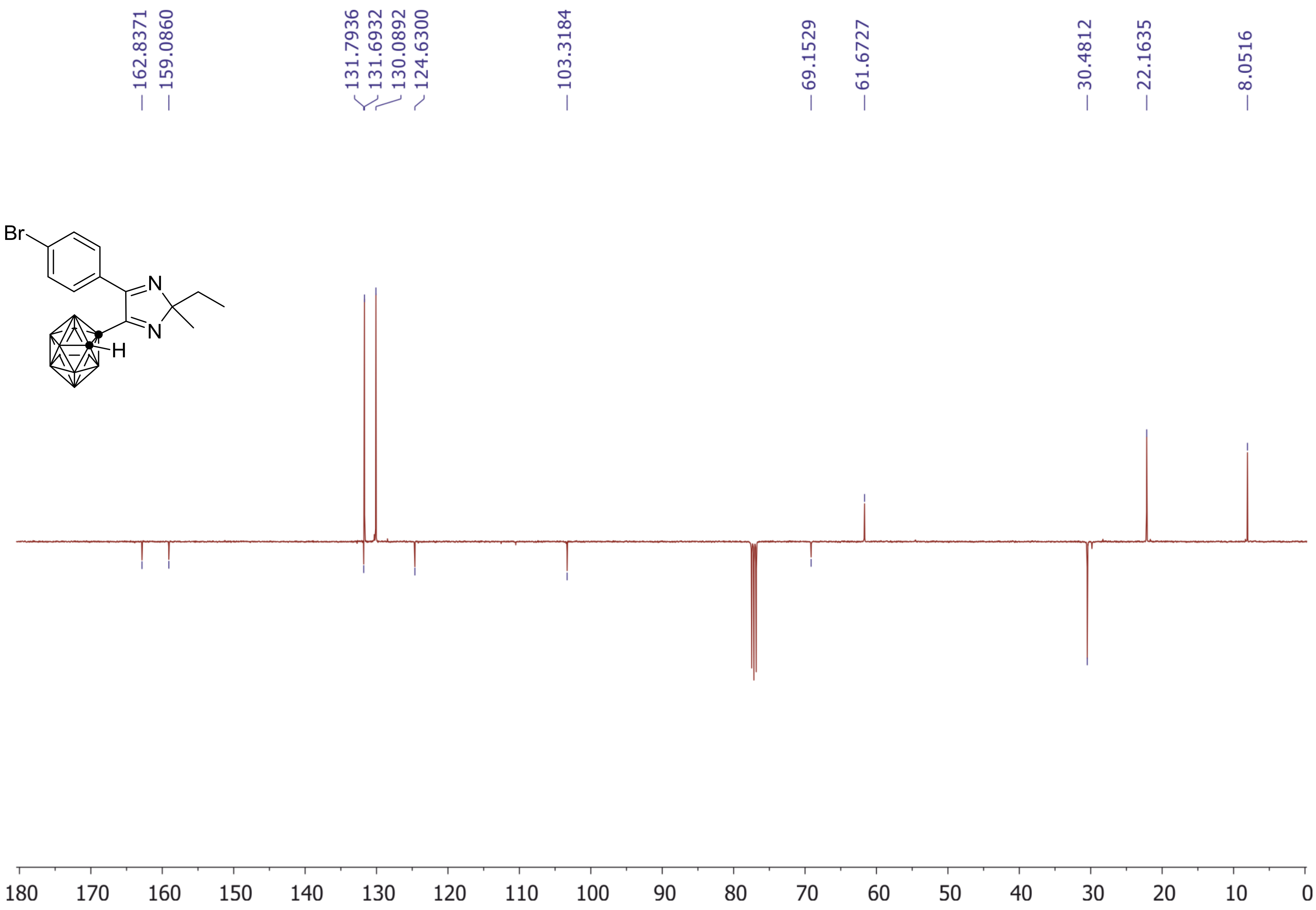

Figure S16. $^{13}C\{^1H\}$ NMR Spectrum (APT) of 1-(5-(4-bromophenyl)-2-ethyl-2-methyl-2*H*-imidazol-4-yl)-1,2-dicarba-*closo*-dodecaborane (**4d**).

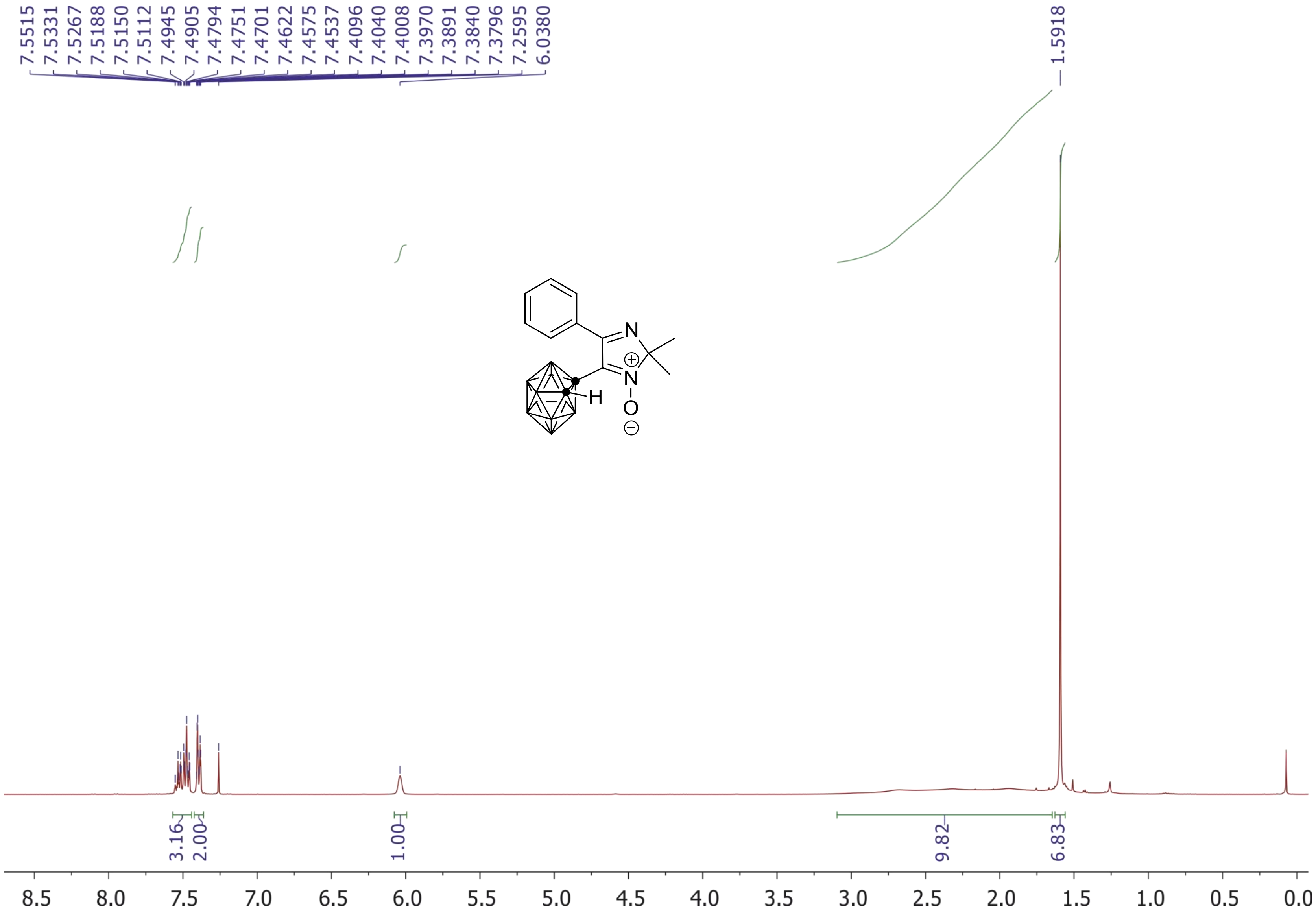

Figure S17. ^{1}H NMR Spectrum of 1-(2,2-dimethyl-1-oxido-4-phenyl-2*H*-imidazol-5-yl)-1,2-dicarba-*closo*-dodecaborane (**5a**).

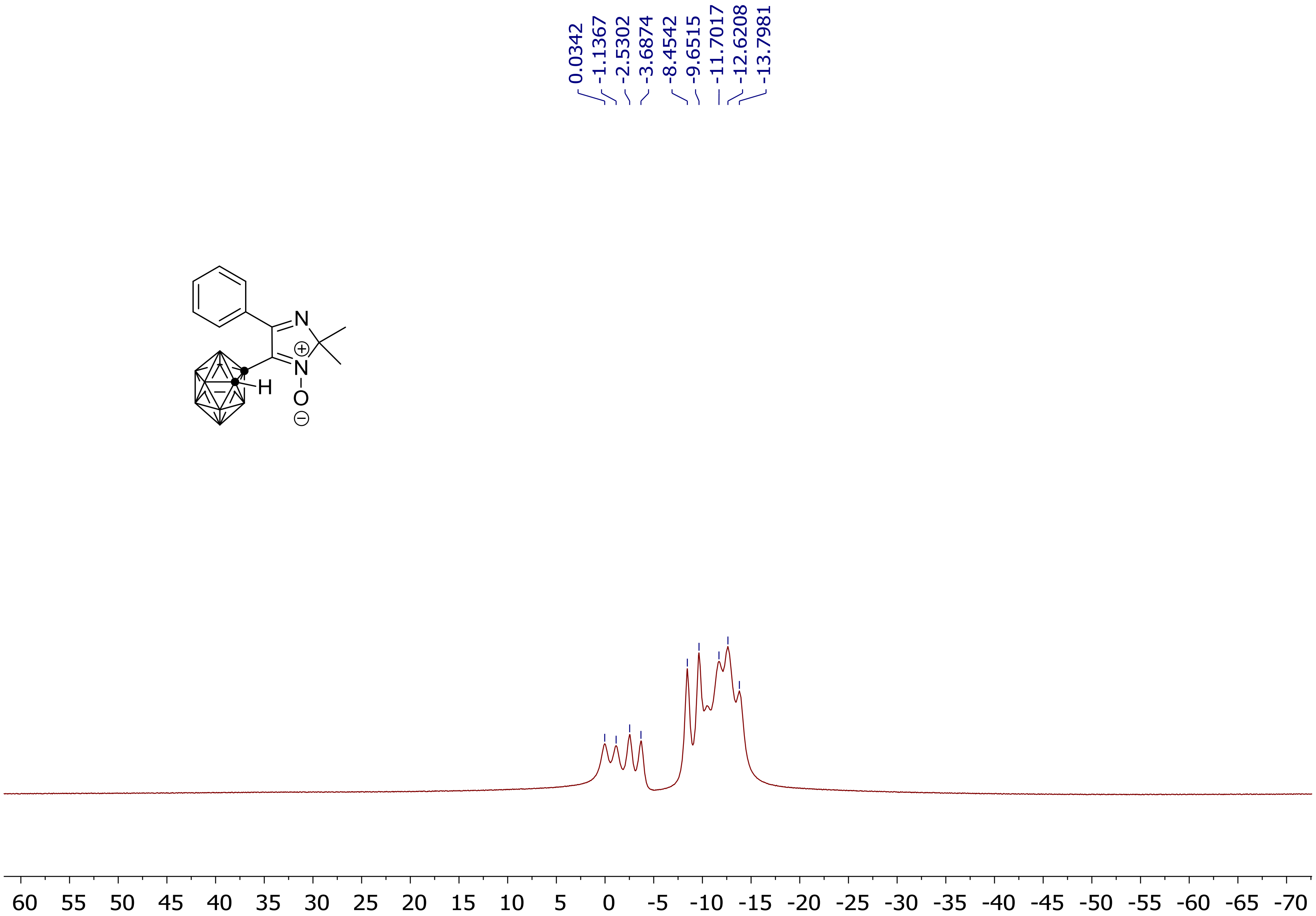

Figure S18. ^{11}B NMR Spectrum of 1-(2,2-dimethyl-1-oxido-4-phenyl-2*H*-imidazol-5-yl)-1,2-dicarba-*closo*-dodecaborane (**5a**).

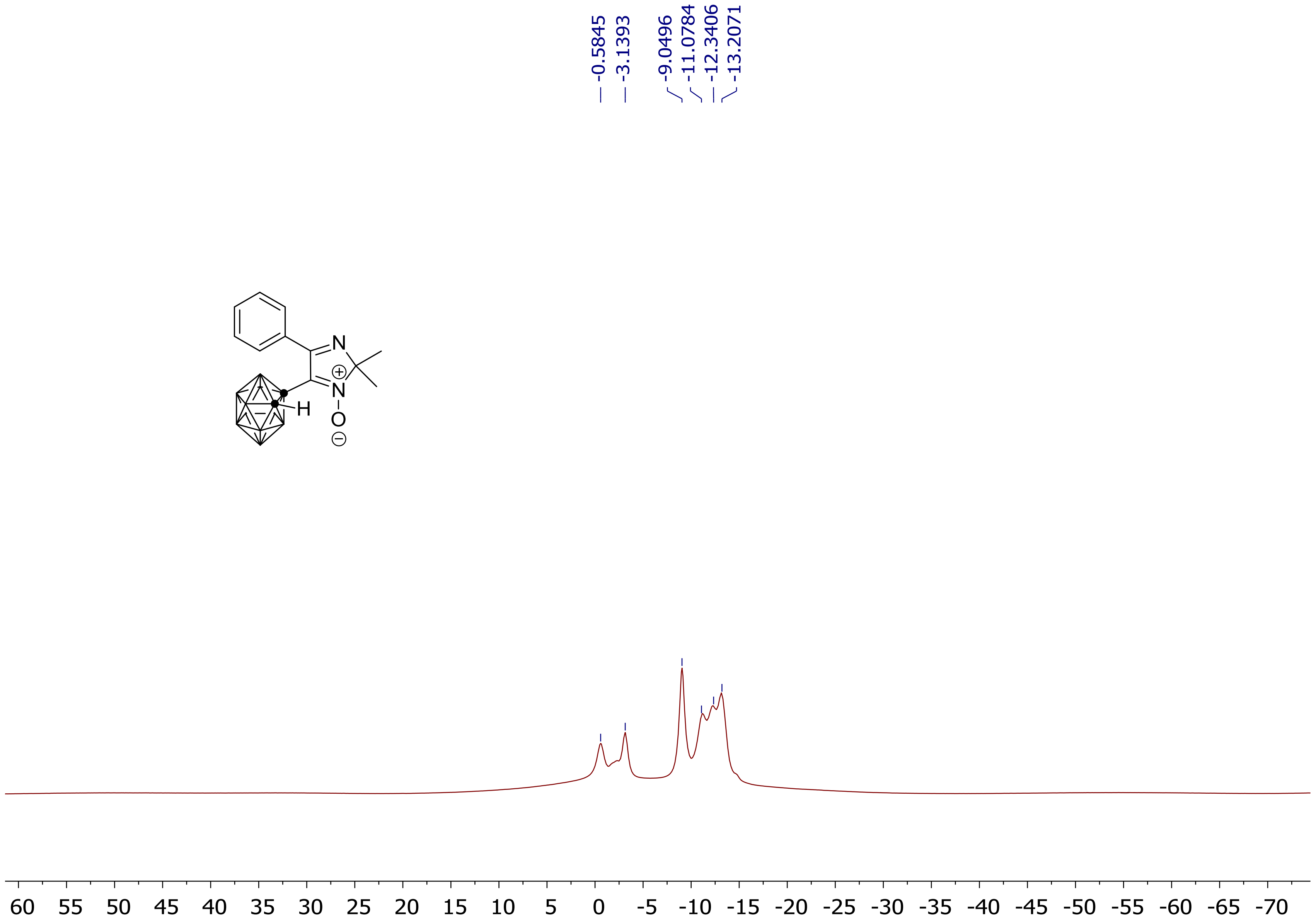

Figure S19. $^{11}B\{^{1}H\}$ NMR Spectrum of 1-(2,2-dimethyl-1-oxido-4-phenyl-2*H*-imidazol-5-yl)-1,2-dicarba-*closo*-dodecaborane (**5a**).

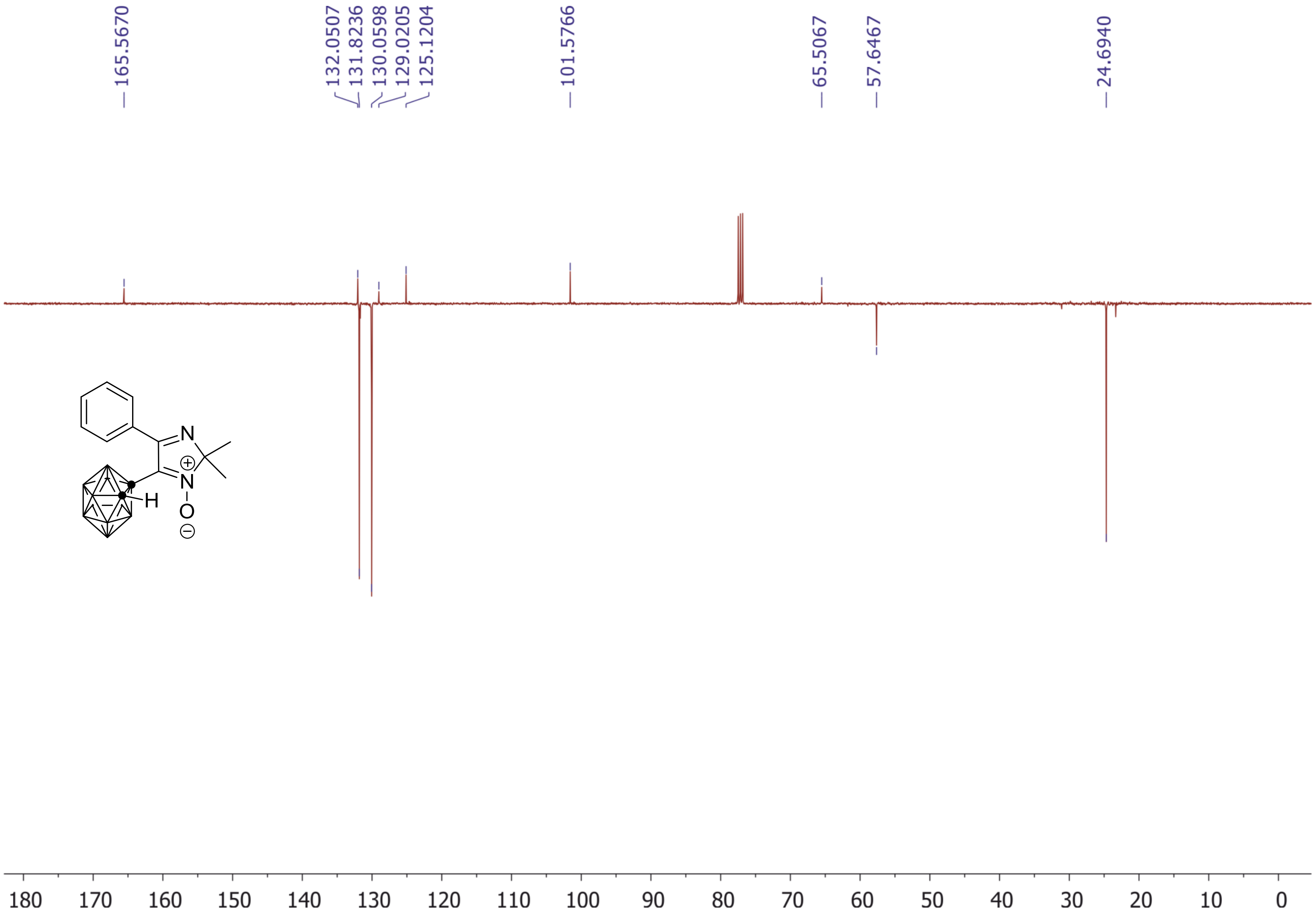

Figure S20. $^{13}C\{^{1}H\}$ NMR Spectrum (APT) of 1-(2,2-dimethyl-1-oxido-4-phenyl-2*H*-imidazol-5-yl)-1,2-dicarba-*closo*-dodecaborane (**5a**).

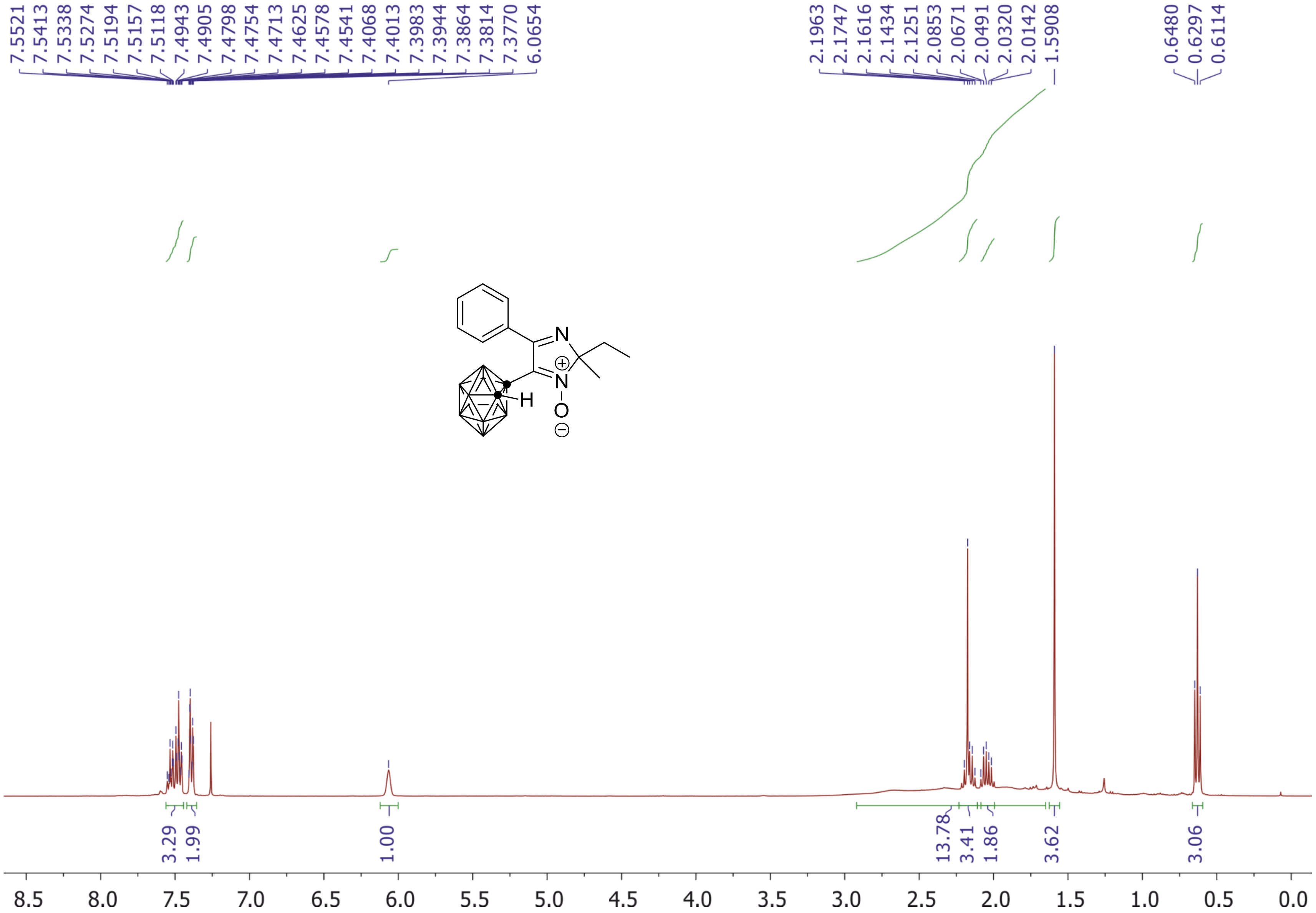

Figure S21. ^{1}H NMR Spectrum of 1-(2-ethyl-2-methyl-1-oxido-4-phenyl-2*H*-imidazol-5-yl)-1,2-dicarba-*closo*-dodecaborane (**5b**).

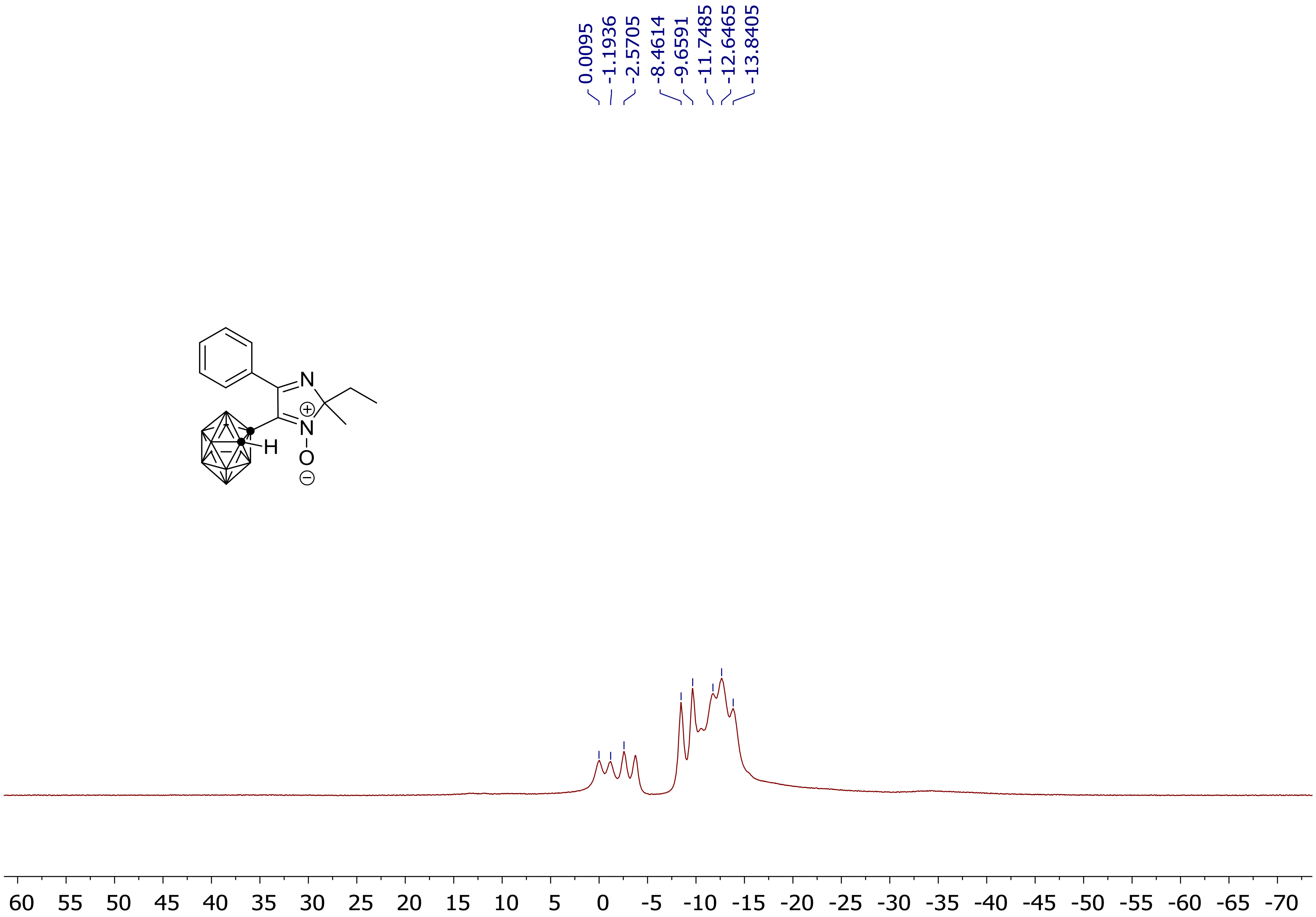

Figure S22. ^{11}B NMR Spectrum of 1-(2-ethyl-2-methyl-1-oxido-4-phenyl-2*H*-imidazol-5-yl)-1,2-dicarba-*closo*-dodecaborane (**5b**).

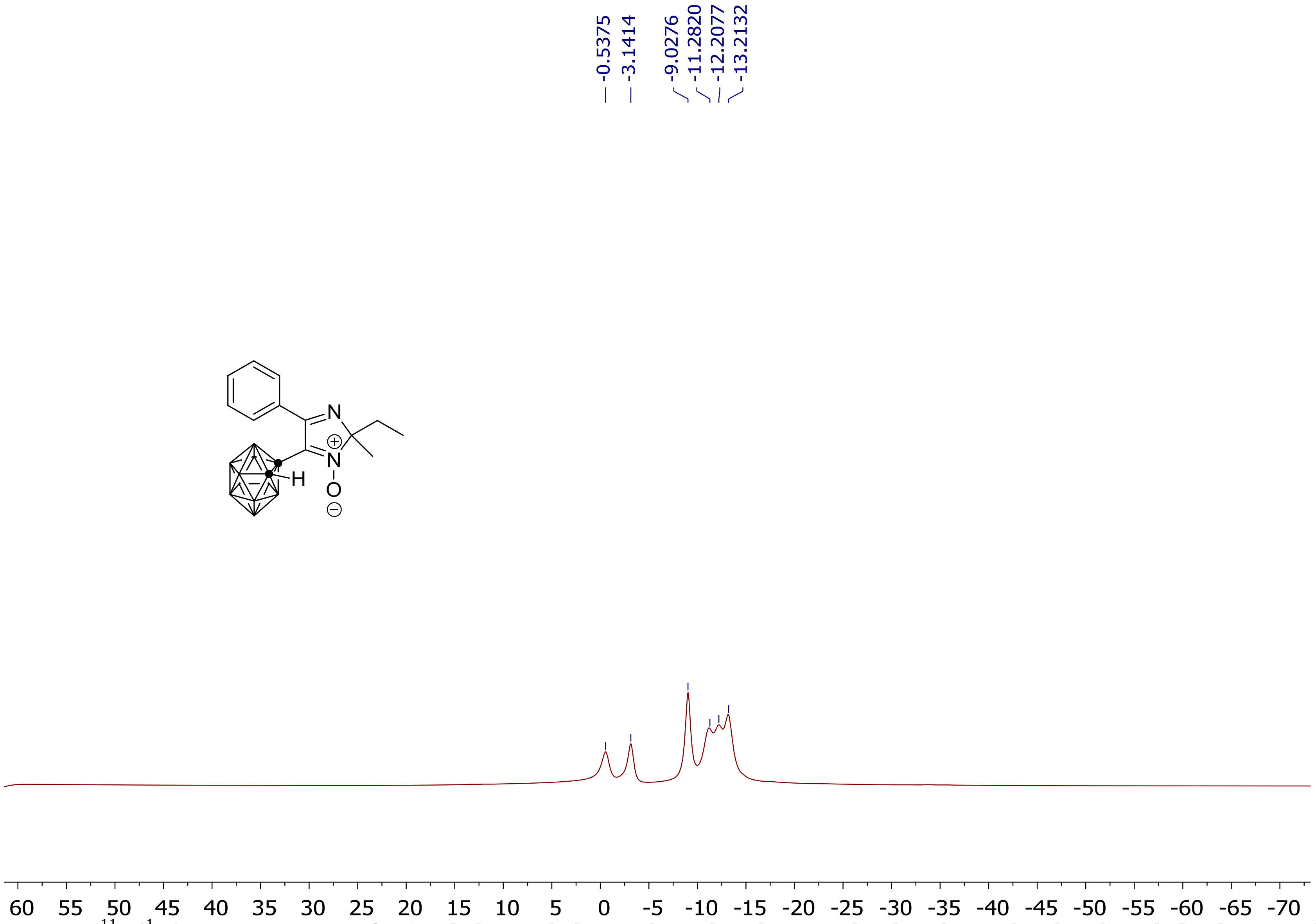

Figure S23. $^{11}B\{^{1}H\}$ NMR Spectrum of 1-(2-ethyl-2-methyl-1-oxido-4-phenyl-2*H*-imidazol-5-yl)-1,2-dicarba-*closo*-dodecaborane (**5b**).

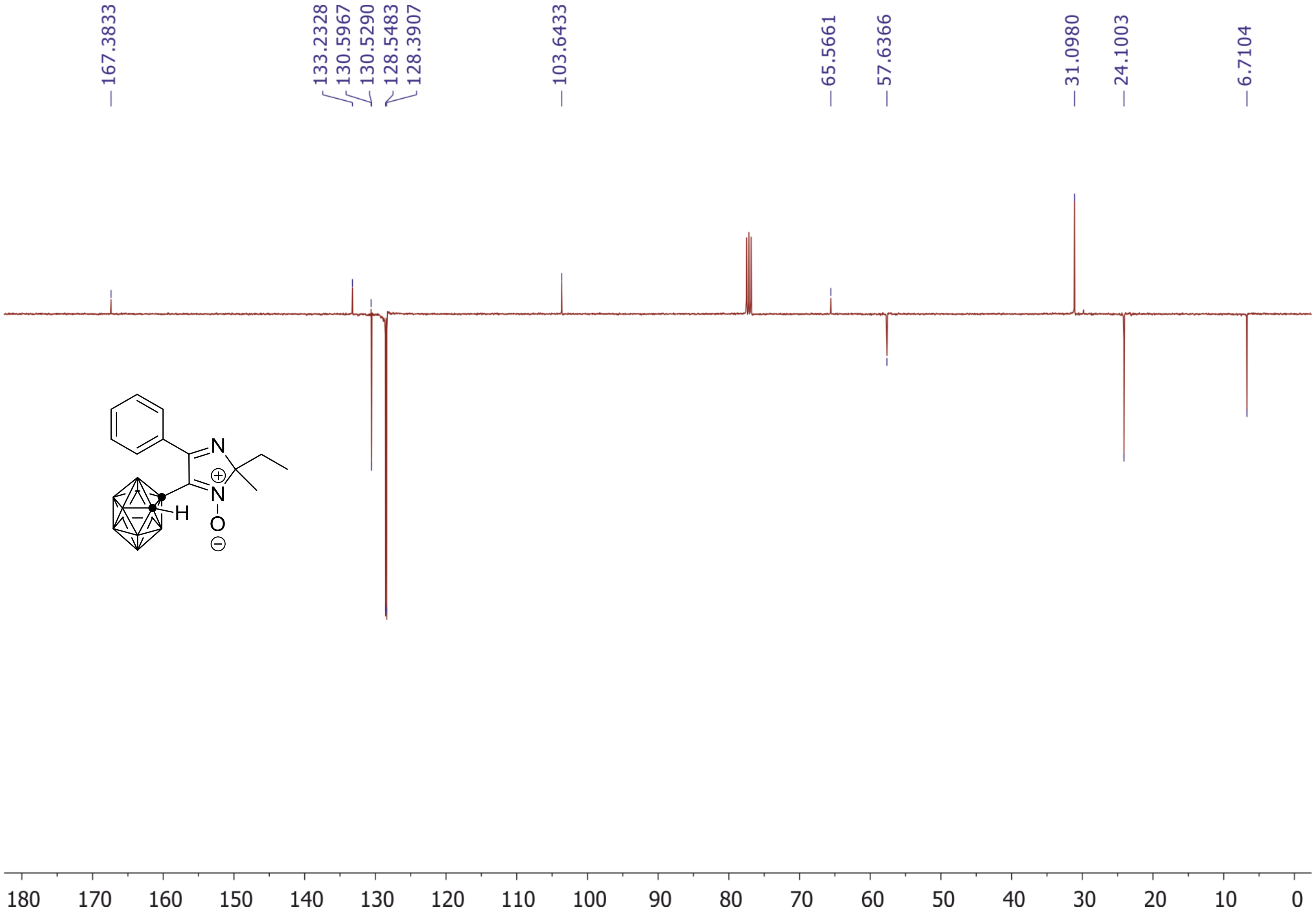

Figure S24. $^{13}C\{^{1}H\}$ NMR Spectrum (APT) of 1-(2-ethyl-2-methyl-1-oxido-4-phenyl-2*H*-imidazol-5-yl)-1,2-dicarba-*closo*-dodecaborane (**5b**).

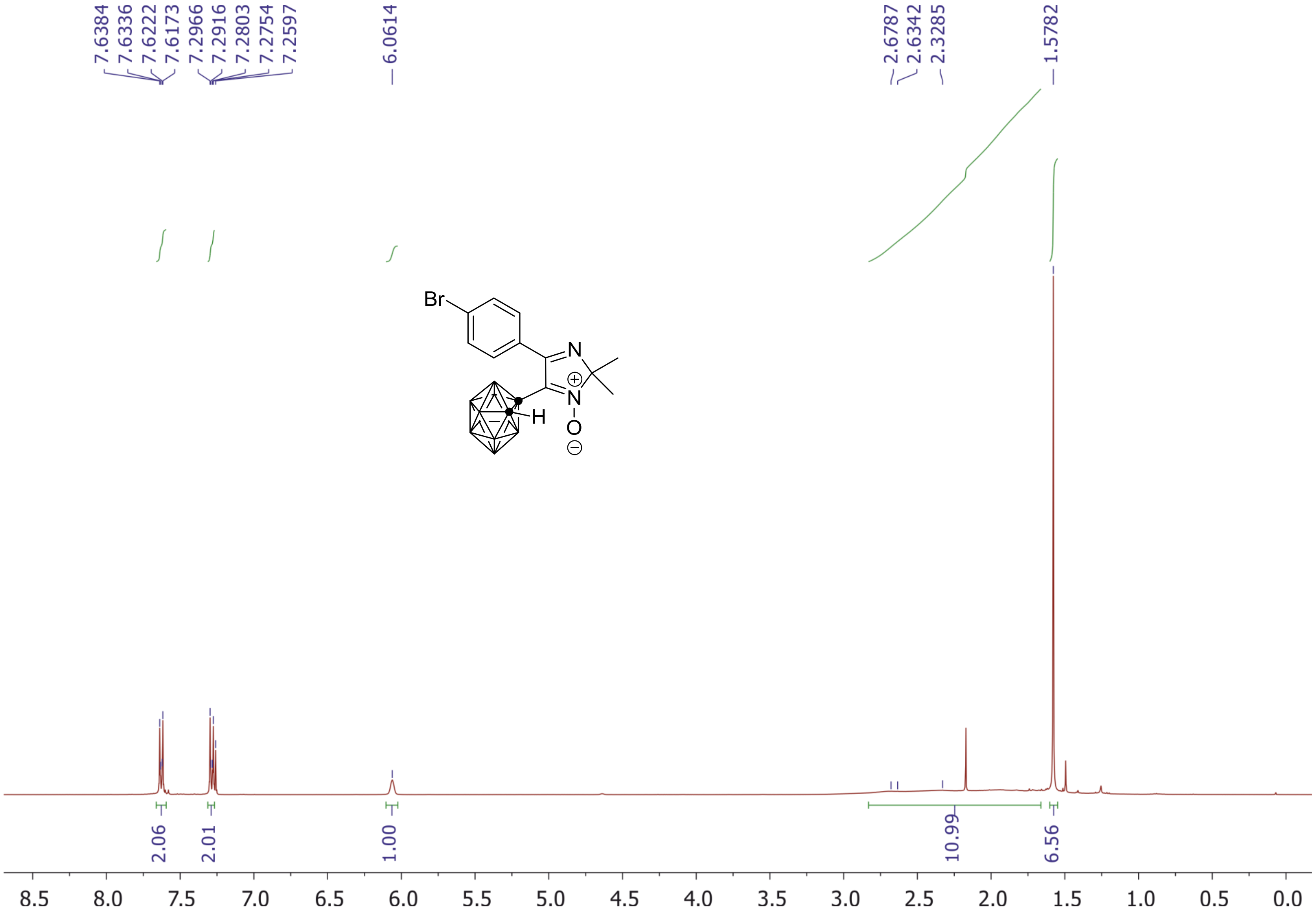

Figure S25. ^{1}H NMR Spectrum of 1-(4-(4-bromophenyl)-2,2-dimethyl-1-oxido-2*H*-imidazol-5-yl)-1,2-dicarba-*closo*-dodecaborane (**5c**).

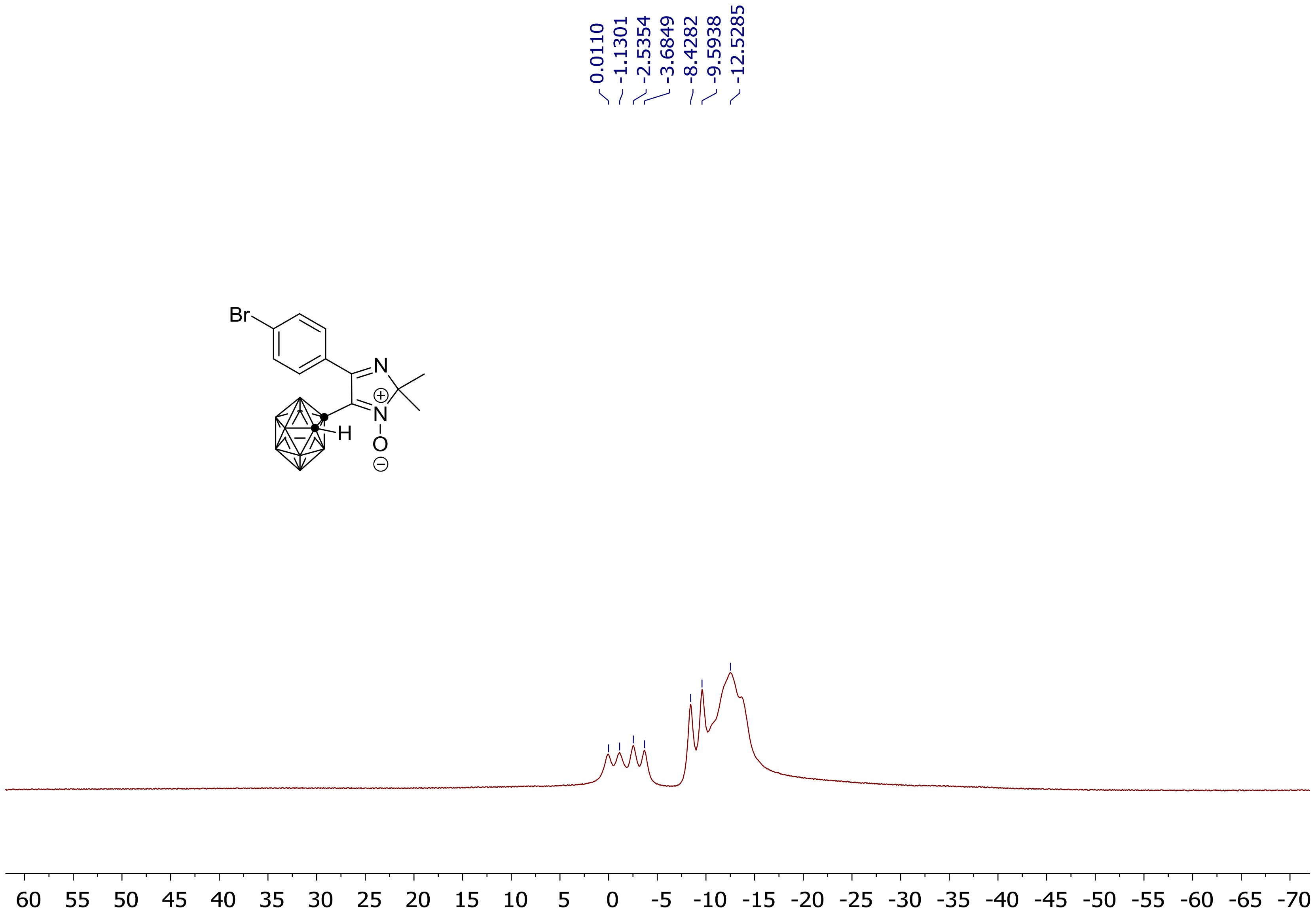

Figure S26. ^{11}B NMR Spectrum of 1-(4-(4-bromophenyl)-2,2-dimethyl-1-oxido-2*H*-imidazol-5-yl)-1,2-dicarba-*closo*-dodecaborane (**5c**).

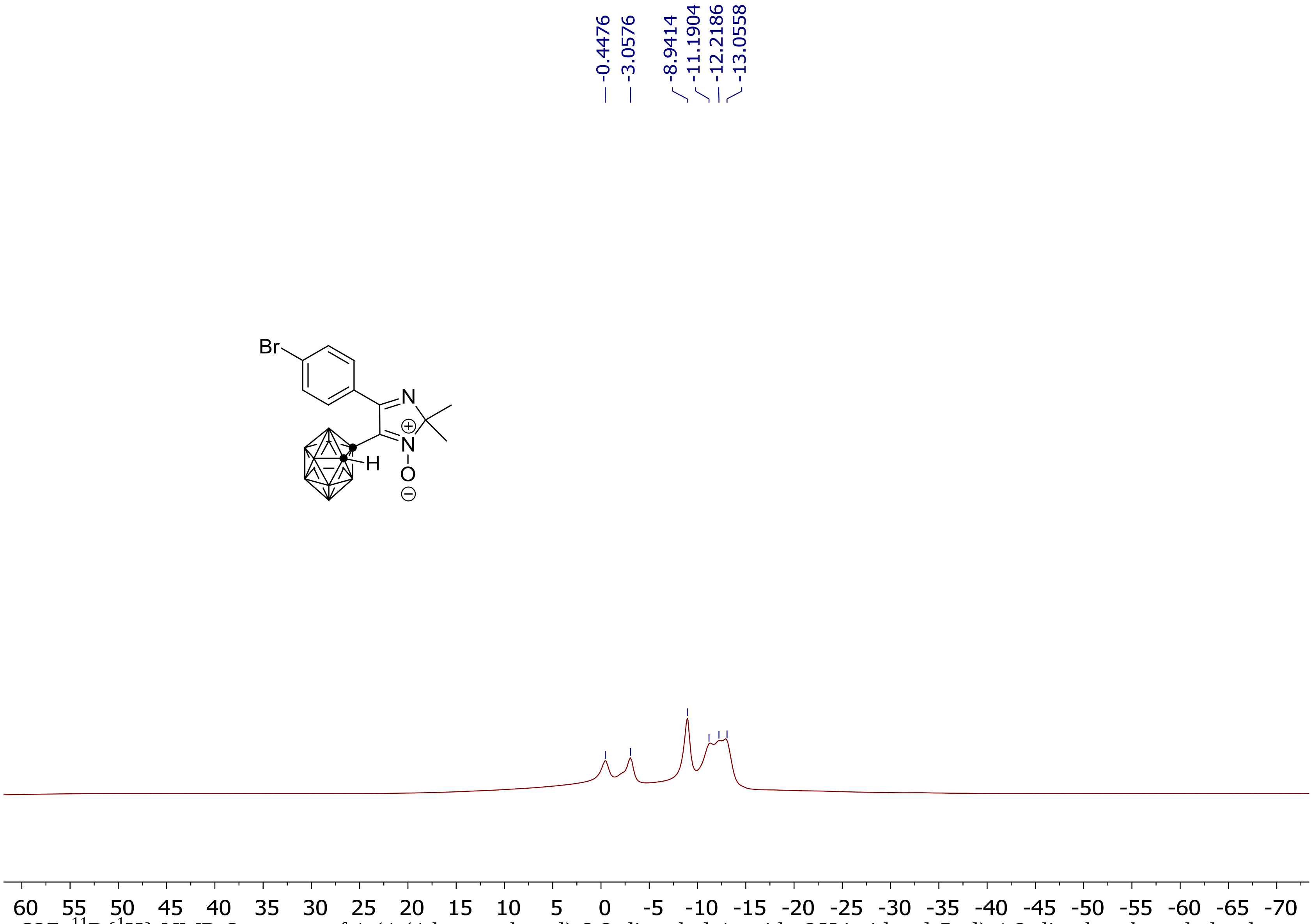

Figure S27. $^{11}B\{^{1}H\}$ NMR Spectrum of 1-(4-(4-bromophenyl)-2,2-dimethyl-1-oxido-2*H*-imidazol-5-yl)-1,2-dicarba-*closo*-dodecaborane (**5c**).

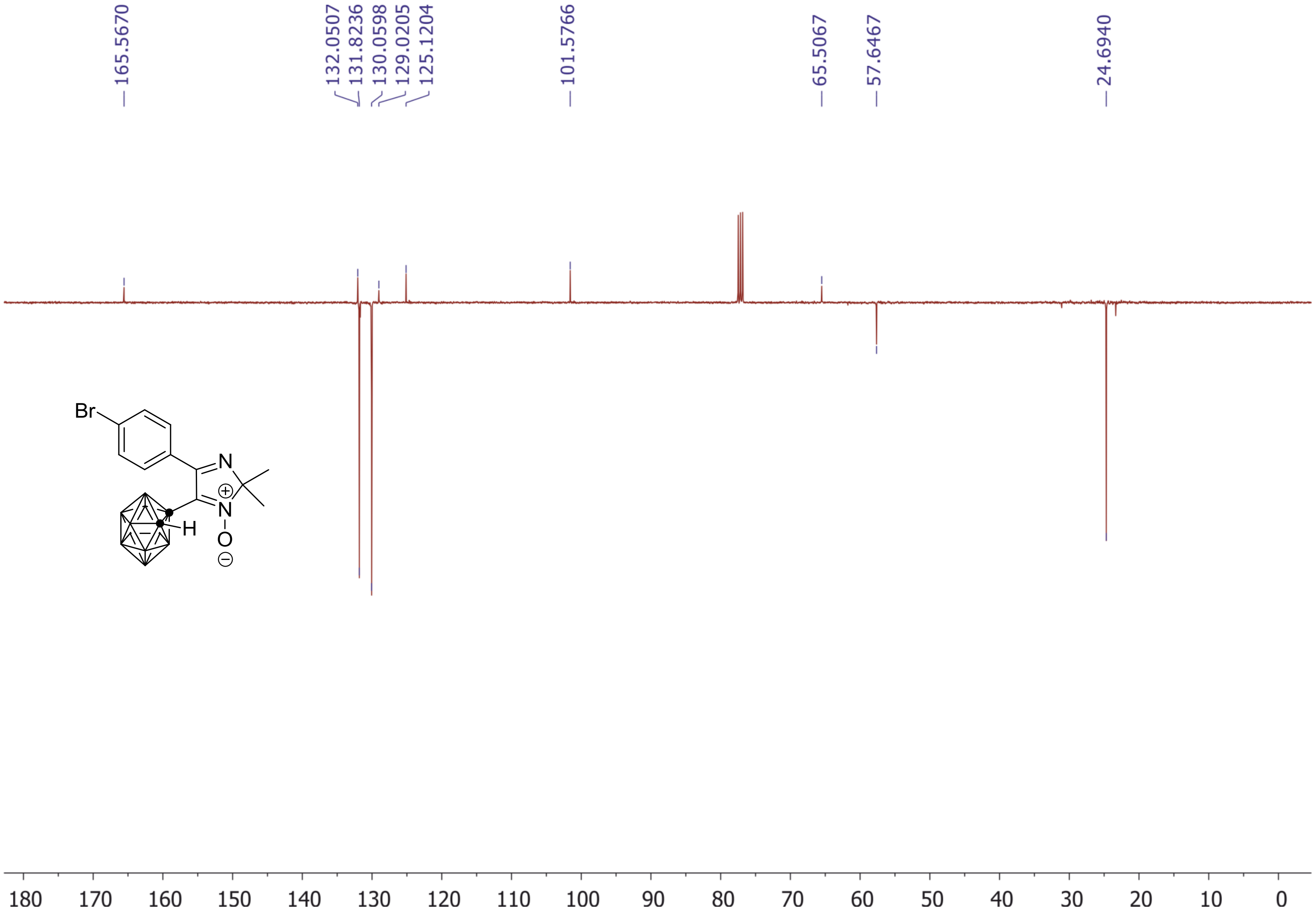

Figure S28. $^{13}C\{^{1}H\}$ NMR Spectrum (APT) of 1-(4-(4-bromophenyl)-2,2-dimethyl-1-oxido-2*H*-imidazol-5-yl)-1,2-dicarba-*closo*-dodecaborane (**5c**).

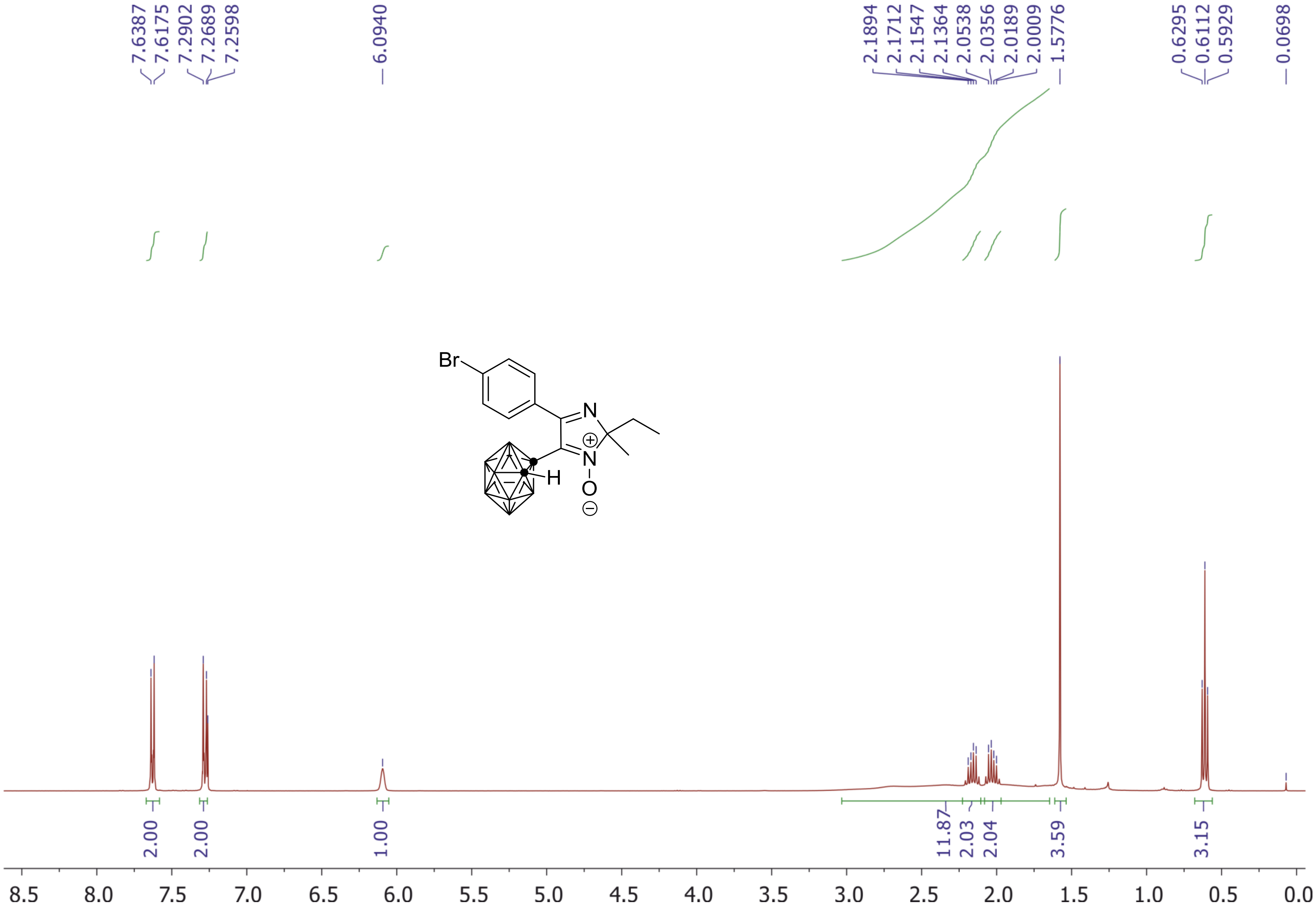

Figure S29. ^{1}H NMR Spectrum of 1-(4-(4-bromophenyl)-2-ethyl-2-methyl-1-oxido-2*H*-imidazol-5-yl)-1,2-dicarba-*closo*-dodecaborane (**5d**).

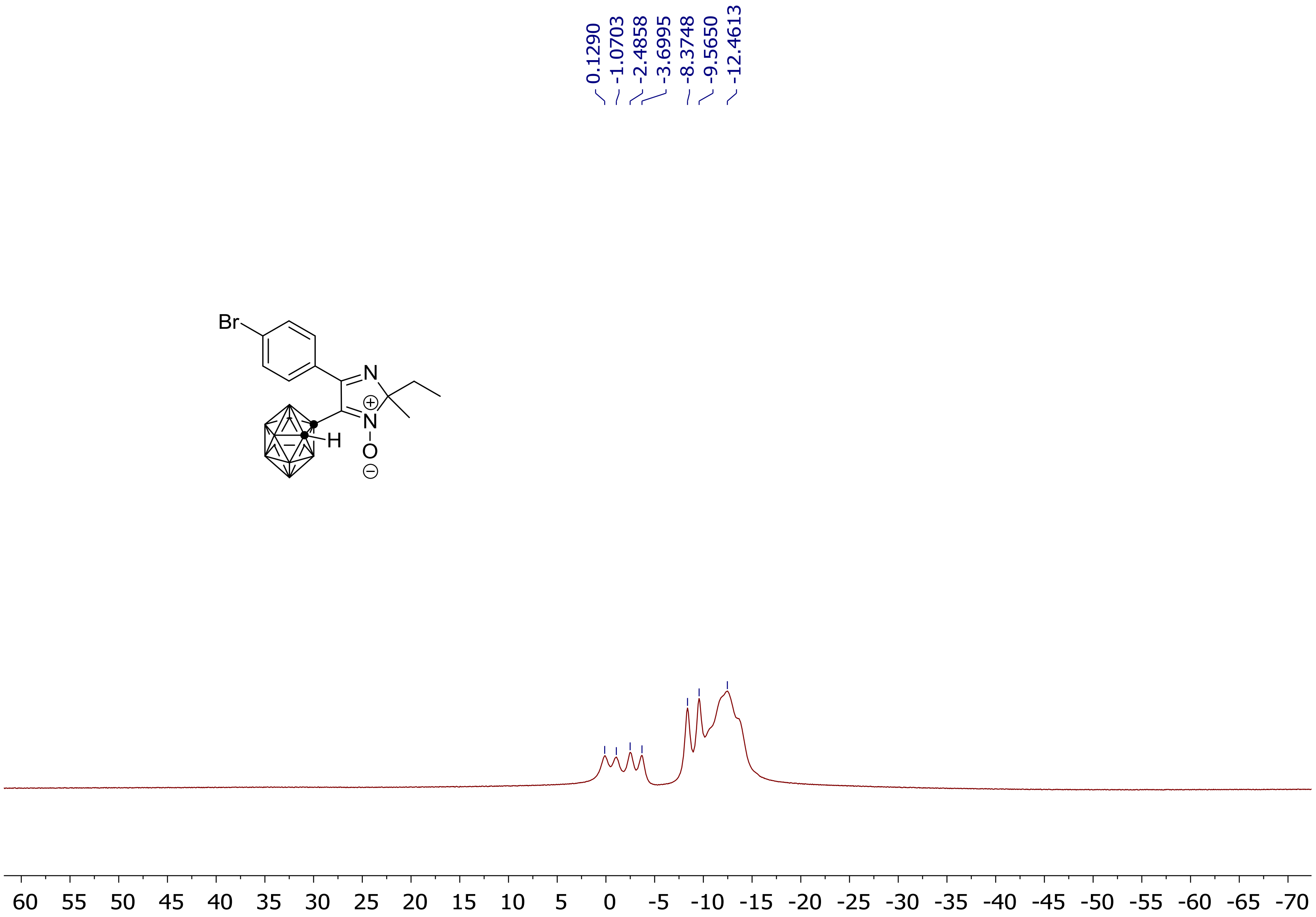

Figure S30. ^{11}B NMR Spectrum of 1-(4-(4-bromophenyl)-2-ethyl-2-methyl-1-oxido-2*H*-imidazol-5-yl)-1,2-dicarba-*closo*-dodecaborane (**5d**).

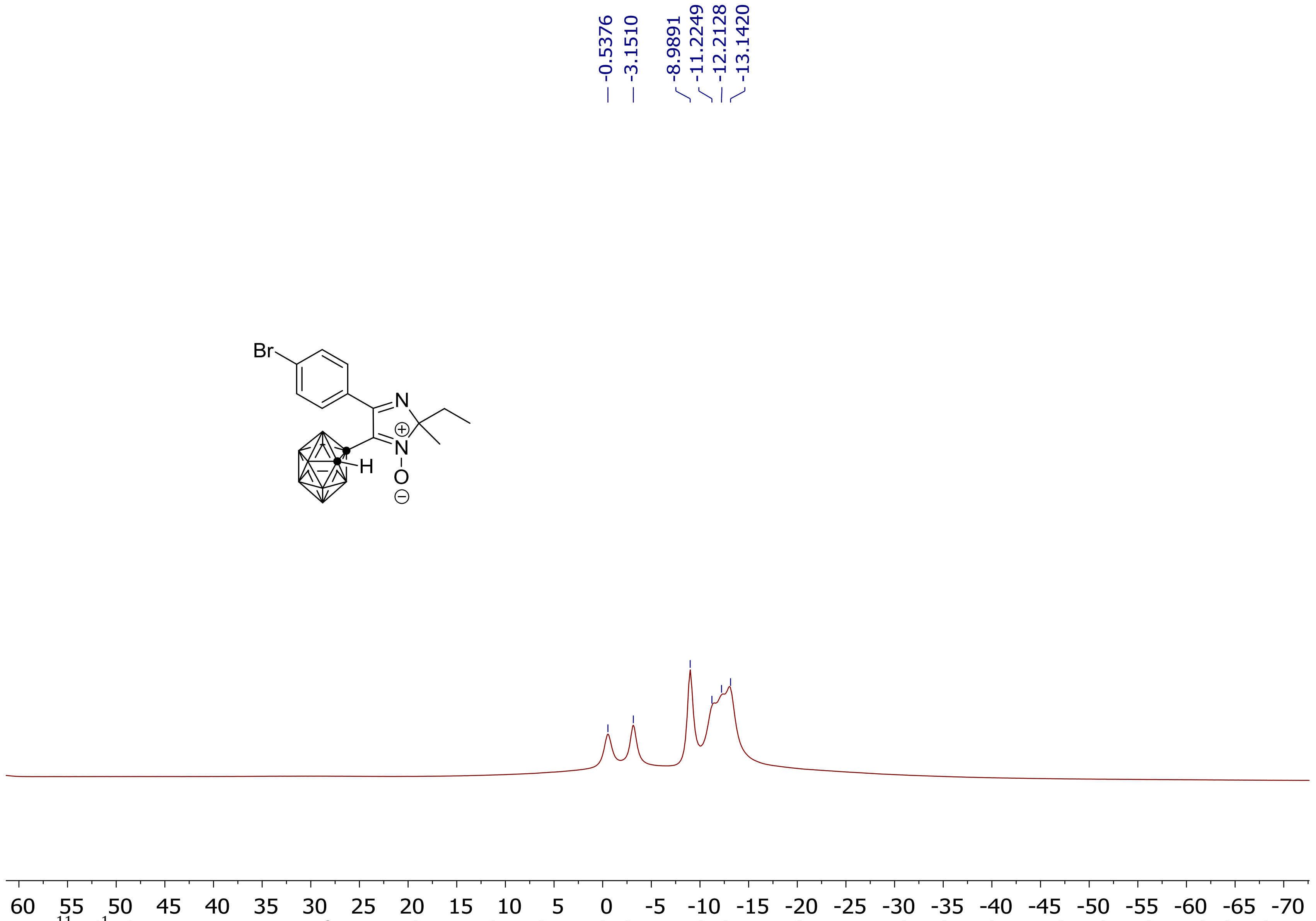

Figure S31. $^{11}B\{^{1}H\}$ NMR Spectrum of 1-(4-(4-bromophenyl)-2-ethyl-2-methyl-1-oxido-2*H*-imidazol-5-yl)-1,2-dicarba-*closo*-dodecaborane (**5d**).

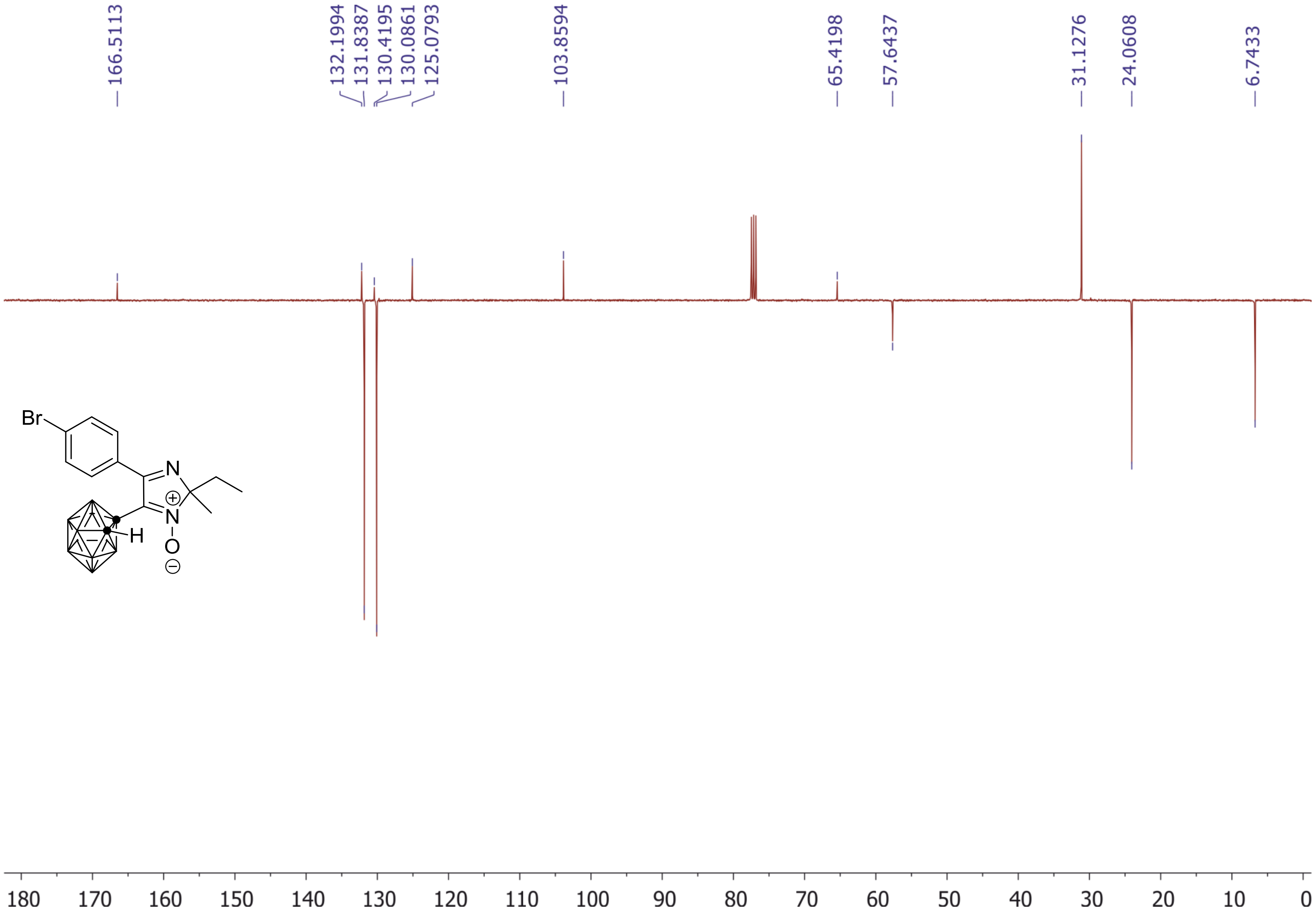

Figure S32. $^{13}C\{^{1}H\}$ NMR Spectrum (APT) of 1-(4-(4-bromophenyl)-2-ethyl-2-methyl-1-oxido-2*H*-imidazol-5-yl)-1,2-dicarba-*closo*-dodecaborane (**5d**).

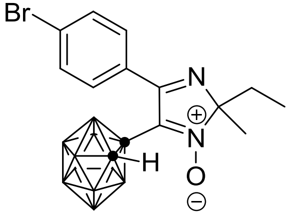

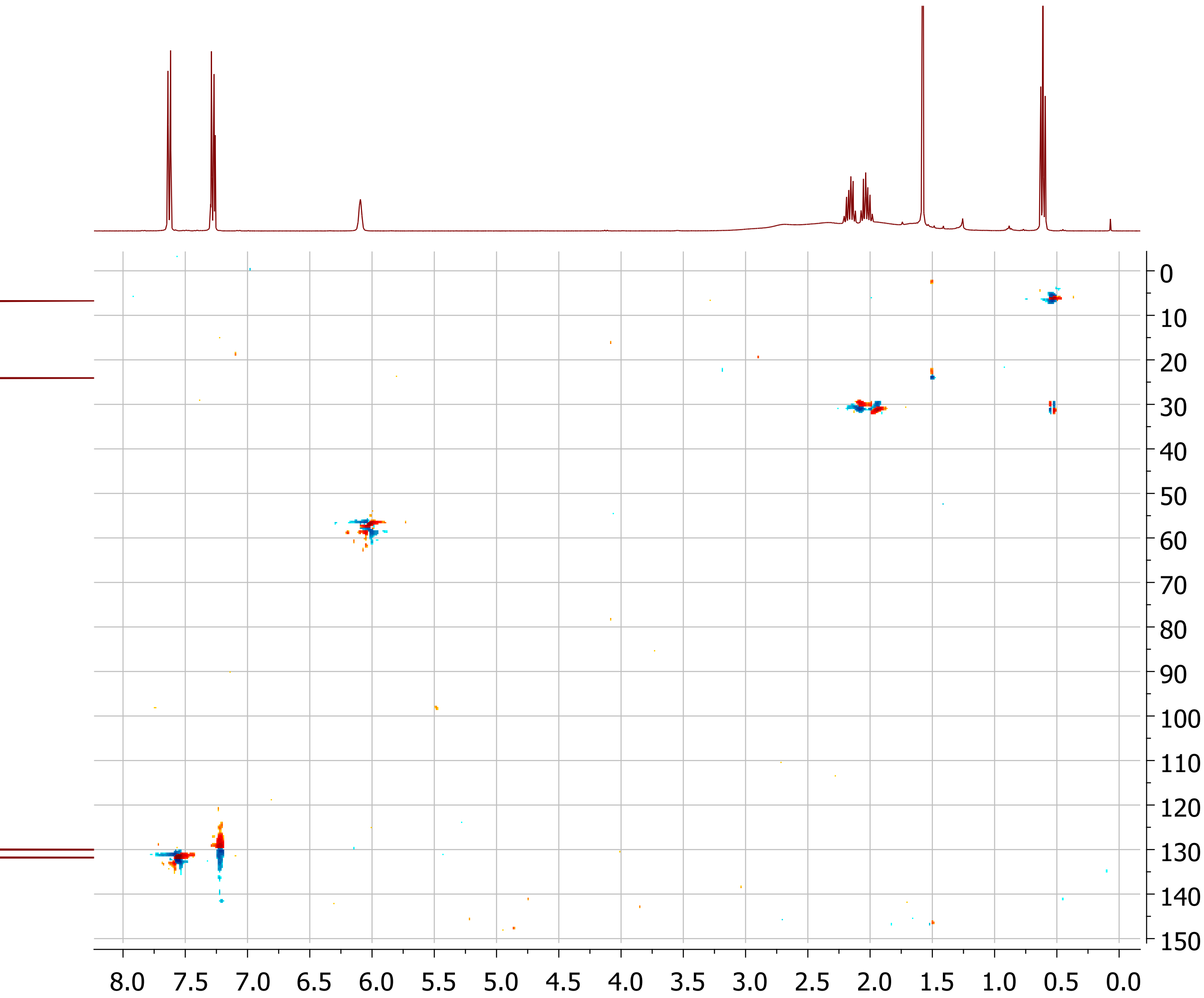

Figure S33. ^{1}H–$^{13}C\{^{1}H\}$ HSQC Spectrum of 1-(4-(4-bromophenyl)-2-ethyl-2-methyl-1-oxido-2*H*-imidazol-5-yl)-1,2-dicarba-*closo*-dodecaborane (**5d**).

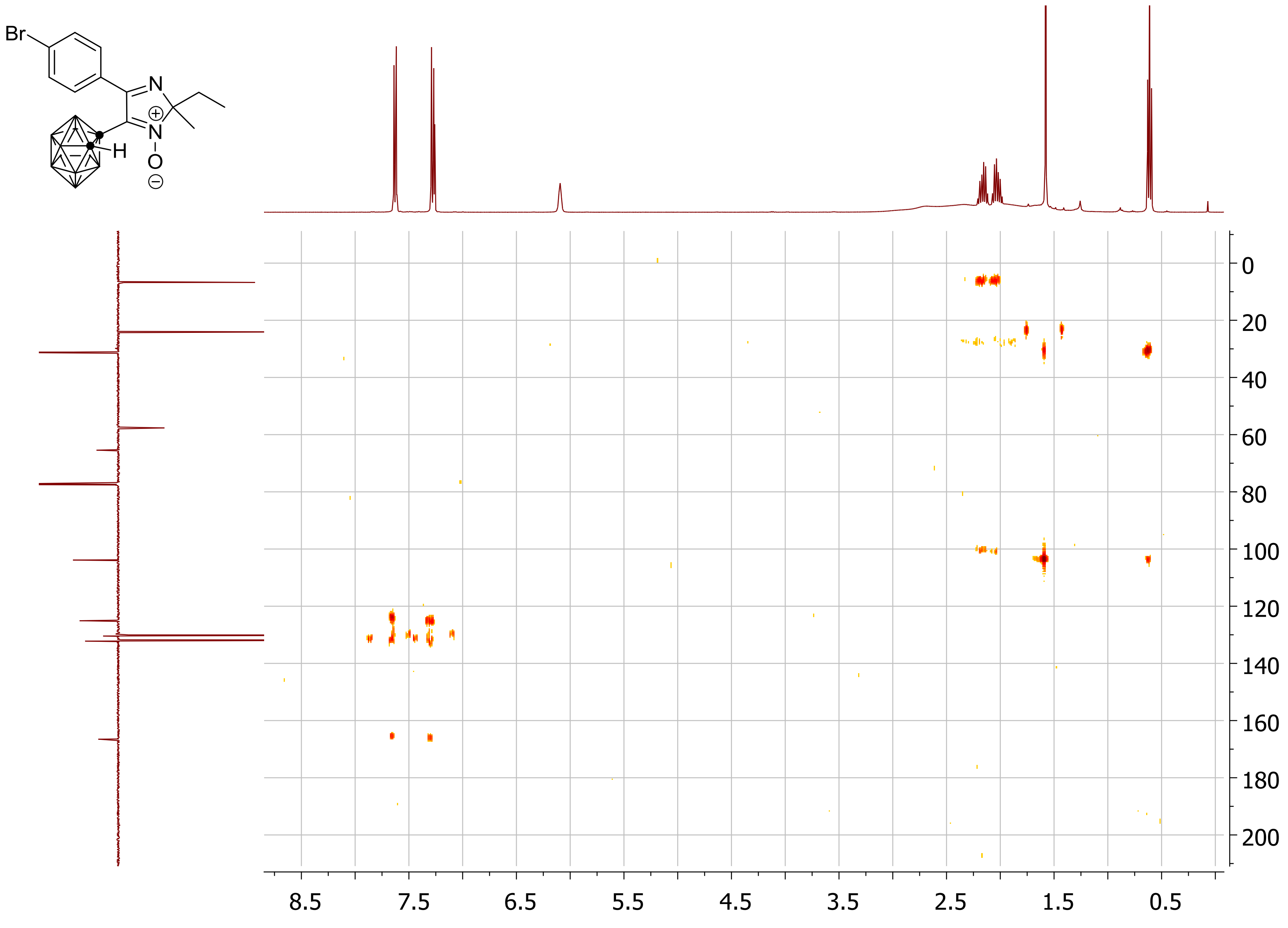

Figure S34. $^{1}H–^{13}C\{^{1}H\}$ HMBC Spectrum of 1-(4-(4-bromophenyl)-2-ethyl-2-methyl-1-oxido-2*H*-imidazol-5-yl)-1,2-dicarba-*closo*-dodecaborane (**5d**).

X-Ray Diffraction Studies

The crystallographic data and basic refinement parameters for **5d** are shown in Table S1.

Table S1. X-ray analysis data and basic refinement parameters for **5d**

Parameter	5d
Molecular formula	$C_{14}H_{23}B_{10}BrN_2O$
Molecular weight	423.35
T/K	295(2)
λ/Å	0.71073
Syngony	monoclinic
Space group	$P12_11$
a/Å	12.4915(8)
b/Å	9.3665(5)
c/Å	18.7577(13)
α/deg	90.00
β/deg	104.277(7)
γ/deg	90.00
V, $Å^3$	2126.9(2)
Z	4
d_{calc}/g·cm^{-3}	1.322
μ/mm^{-1}	1.940
F(000)	856
Crystal size/mm	0.43x 0.36x 0.22
2θ-Scan range/deg	3.55-30.89
Completeness based on $2\theta_{мax}$	0.7263
Completeness based on 2θ= 52°	0.9859
hkl ranges	-16 < h < 16<br>-7 < k < 12<br>-24 < l < 26
Total number of reflections	15307
Number of independent reflections	9757
Number of reflections with $I > 2\sigma(I)$	6070
Number of refined parameters	552
Absorption correction	multi-scan
GOOF (based on F^2)	1.006
R factors (based on reflections with $I > 2\sigma(I)$)	
R_1	0.0668
wR_2	0.1677
R factors (based on all reflections)	
R_1	0.1161
wR_2	0.2103
$\Delta\rho_{max}$/ $\Delta\rho_{min}$, e$Å^{-3}$	0.759/ -0.546

Crystallographic data (excluding structure factors) for the structures **5d** in this paper have been deposited with the Cambridge Crystallographic Data Centre as supplementary publication no. CCDC 1855797. Copy of the data can be obtained, free of charge, on application to CCDC, 12 Union Road, Cambridge CB2 1EZ, UK, (fax: +44(0)1223 336033 or e-mail: deposit@ccdc.cam.ac.uk).

References

[1] Sheldrick, G. M. *Acta Crystallorg., Sect. A* **2008**, 64, 112-122.

doi: 10.1107/S0108767307043930

[2] Clark, R. C.; Reid, J. S. *Acta Crystallorg., Sect. A* **1995**, 51, 887-897.

doi: 10.1107/S0108767395007367

[3] Kirilyuk, I. A.; Grigor'ev, I. A.; Volodarskii, L. B. *Russ. Chem. Bull*. **1991**, 40, 1871-1879.

doi: 10.1007/BF00960421

[4] Varaksin, M. V.; Utepova, I. A.; Chupakhin, O. N.; Charushin, V. N. *J. Org. Chem*. **2012**, 77, 9087- 9093.

doi: 10.1021/jo301618b

[5] Galliamova, L. A.; Varaksin, M. V.; Chupakhin, O. N.; Slepukhin, P. A.; Charushin, V. N. *Organomatallics* **2015**, 34, 5285−5290

doi: 10.1021/acs.organomet.5b00736
